# Substrate and enzyme determinants for recognition by human mitochondrial RNase P

**DOI:** 10.1093/nar/gkaf1145

**Published:** 2025-11-20

**Authors:** Enxhi Hazisllari, Danijela Radovanović, Ursula Toth, Elisa Vilardo, Roland K Hartmann, Walter Rossmanith

**Affiliations:** Center for Anatomy & Cell Biology, Medical University of Vienna, 1090 Vienna, Austria; Center for Anatomy & Cell Biology, Medical University of Vienna, 1090 Vienna, Austria; Center for Anatomy & Cell Biology, Medical University of Vienna, 1090 Vienna, Austria; Center for Anatomy & Cell Biology, Medical University of Vienna, 1090 Vienna, Austria; Institute of Pharmaceutical Chemistry, Philipps-University Marburg, 35037 Marburg, Germany; Center for Anatomy & Cell Biology, Medical University of Vienna, 1090 Vienna, Austria

## Abstract

RNase P enzymes of widely varying architectures recognize the 5′-leader/acceptor-stem junction and the D/T loop-interaction region of precursor tRNAs to direct cleavage to the 5′ end of tRNAs. In contrast, human mitochondrial RNase P (mtRNase P) encases the entire tRNA with the aid of the methyltransferase subcomplex TRMT10C–SDR5C1. Here, we performed a kinetic analysis of substrate recognition by mtRNase P using substrate and protein variants. Surprisingly, processing by mtRNase P was found to be more efficient for tRNA precursors with longer 5′ extensions and decreased sharply at a leader length of 1 nt. MtRNase P also employs a more rigid “measuring mechanism” for cleavage-site selection than the related single-subunit enzymes, so that even substrates with a G:C base-pair extension of the acceptor stem are cleaved predominantly at the canonical site. The specific contacts of TRMT10C–SDR5C1 with the anticodon loop are not crucial for efficient processing, but without interactions with the pre-tRNA, TRMT10C–SDR5C1 is unable to stimulate cleavage by the nuclease subunit PRORP, also explaining why mtRNase P reaches its limits with the D-armless mitochondrial tRNA^Ser(AGY)^. Our findings set human mtRNase P apart in terms of substrate recognition from all other known forms of RNase P, including the related single-polypeptide PRORPs.

## Graphical Abstract

**Graphical Abstract:**
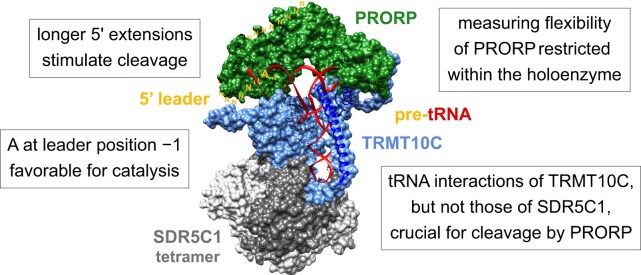

## Introduction

Ribonuclease P (RNase P) is the essential endonuclease responsible for the removal of the 5′-leader sequences from precursor transfer RNAs (pre-tRNAs) in all domains of life [1]. Initially discovered in Bacteria, this enzyme is frequently found as a ribonucleoprotein (RNP) enzyme, composed of a catalytic RNA subunit and one or more protein cofactors, whose number apparently increased with the complexity of the organism [1–6]. An alternative form of RNase P, typically consisting of a single ∼60 kDa polypeptide, was identified in many Eukarya and named proteinaceous RNase P (PRORP) [1, 7–10]; in addition to the eukaryal PRORP enzyme, a distinct type of protein-only RNase P, comprising solely a metallonuclease domain with a small inserted dimerization domain, was discovered in some bacteria and archaea, and referred to as homolog of *Aquifex aeolicus* RNase P (HARP) [10–12]. All eukaryotic PRORPs share a characteristic Λ-shaped tripartite domain architecture, consisting of an N-terminal pentatricopeptide repeat (PPR) domain, a split zinc-binding domain, and a C-terminal PIN-like nuclease domain [10, 13]. While in most eukarya PRORPs work as single-subunit enzymes, human mitochondrial PRORP (aka MRPP3) requires two additional proteins for its function: tRNA methyltransferase 10C (TRMT10C aka MRPP1) and short-chain dehydrogenase/reductase family C member 1 (SDR5C1 aka MRPP2 or HSD17B10) [7, 10, 14, 15].

TRMT10C is a member of the SpoU-TrmD (SPOUT) enzyme superfamily and is responsible for the *N*^1^-methylation of purines at position 9 (m^1^R9) of mitochondrial tRNAs. Unlike its cytosolic and archaeal homologs that function as monomers, TRMT10C requires an additional protein called SDR5C1 for efficient activity [14]. SDR5C1 is a dehydrogenase involved in the penultimate step of the β-oxidation of branched- and short-chain fatty acids as well as isoleucine [16, 17]. This 27-kDa polypeptide forms a symmetric homo-tetramer, with each monomer containing a dehydrogenase active site able to bind the NADH cofactor [18, 19]. The symmetrical structure of SDR5C1 leads to the formation of two identical binding grooves on opposite sides. These structural features are consistent with the potential simultaneous binding of TRMT10C molecules to each side, resulting in a 4:2 stoichiometry between SDR5C1 and TRMT10C [7, 10, 14, 20–23]. Interestingly, the role of SDR5C1 in the context of TRMT10C methylation or endonuclease activity of human PRORP appears to be primarily that of a scaffold. Indeed, neither the methyltransferase activity of TRMT10C nor the dehydrogenase activity of SDR5C1 directly impact the cleavage of pre-tRNA by PRORP; nevertheless, both proteins, SDR5C1 and TRMT10C, are required for efficient PRORP activity [14, 15]. Remarkably, PRORP exhibits limited cleavage activity on some mitochondrial pre-tRNAs on its own, but with reduced efficiency compared to the holoenzyme including TRMT10C–SDR5C1 [15].

The architecture of the active site and the overall geometry of human PRORP appear to resemble that of (single-subunit) PRORPs from *Arabidopsis thaliana* [22, 24, 25]. The recent cryo-electron microscopy (cryo-EM) structure of human mitochondrial RNase P (mtRNase P), in complex with pre-tRNA, unveiled that an arch-like shaped PRORP binds on top of the pre-tRNA–TRMT10C–SDR5C1 complex, interacting with both, the pre-tRNA and TRMT10C, via its nuclease and PPR domains. TRMT10C encases part of the tRNA by means of two tRNA-interacting domains connected by an adapter loop and helix; the latter is anchored to a groove at the surface of the SDR5C1 tetramer. While TRMT10C establishes interactions with all four subdomains of the tRNA, SDR5C1 seems to form contacts with the anticodon (AC) loop only [22, 23].

Human mitochondria harbor a distinct set of tRNAs, which differ from canonical tRNAs [26–28]. Specifically, they exhibit a strong bias in nucleotide content toward A, U, and C-rich sequences, which has implications for their stability and folding. In addition, mitochondrial tRNAs display significant variability in D and T loop lengths, and many of them lack some or all of the conserved and semi-conserved motifs that are critical for the formation of the tRNA “elbow” region. Because the “elbow,” along with the acceptor arm, is involved in interactions with RNA-based bacterial RNase P and *A. thaliana* PRORP [10, 25, 29–33], the absence of these tertiary interaction motifs in many mitochondrial tRNAs suggests a distinct recognition mechanism specific for human mtRNase P.

Here, we studied structural and sequence requirements for efficient processing by human mtRNase P. We performed enzyme kinetics experiments using a variety of altered and truncated pre-tRNA variants to identify key determinants of substrate recognition and to define the minimal structural requirements for cleavage by mtRNase P. Some of the protein–protein and protein–tRNA interactions involving TRMT10C and suggested to be crucial based on the recent cryo-EM structure [22] were evaluated by kinetic analyses using TRMT10C variants with strategic amino acid changes or deletions. Our results provide mechanistic insights into the molecular interplay between mtRNase P and its pre-tRNA substrates, and contribute to understanding the role of the substrate’s structural elements and the functional domains of the enzyme complex in the cleavage process. Thereby, these results help to understand how mtRNase P manages to cope with the structural diversity of mitochondrial tRNAs.

## Materials and methods

### Expression and purification of recombinant proteins

Recombinant proteins, including C-terminally His-tagged human PRORP, N-terminally His-tagged SDR5C1 and C-terminally His-tagged *A. thaliana* PRORP3 (*At*PRORP3) were produced and purified as previously described [14, 32]. The TRMT10C–SDR5C1 complex was also prepared as previously detailed [14, 15]. Purity and concentrations of the recombinant proteins were determined as recently described [15]; in the case of the purified TRMT10C–SDR5C1 complex, the indicated concentrations refer to the concentration of TRMT10C monomers.

TRMT10C variants Δα1, N126A/K129A, Δα1-N126A/K129A, and Kα3A were generated using the QuikChange site-directed mutagenesis protocol (Agilent Technologies; mutagenesis primers listed in Supplementary Table S1). Native TRMT10C and its variants were expressed in *Escherichia coli* Rosetta2 (DE3) and complexes with recombinant SDR5C1 were purified in parallel with magnetic beads (Dynal) as previously detailed [14]. The purity, stoichiometry and concentration of the purified proteins was assessed by sodium dodecyl sulfate-polyacrylamide gel electrophoresis (SDS–PAGE), Coomassie brilliant blue staining and ImageQuant TL (Cytiva) analysis. All protein concentrations were calculated in reference to a previous TRMT10C–SDR5C1 preparation, where the concentration had been assessed relative to bovine serum albumin [15].

### Precursor tRNA substrates

The *Thermus thermophilus* (*Tt*) pre-tRNA^Gly^ and its leader-length variants, ranging from 14 to 1 nt, the 14-nt leader variant without a trailer, as well as variants with A_−1_, G_−1_, U_−1_, A_73_, and U_−1_-A_72_ were previously described [32]. Leader-length variants, as well as variants with C_−1_, G_−1_, A_−1_, A_1_, and G_−1_-C_73_ of human mitochondrial pre-tRNA^Ala^ were generated by (overlap-extension) polymerase chain reaction (PCR) using plasmids phA1 (encoding human mitochondrial pre-tRNA^Ala^ with flanking sequences) [15] and phYCNA1 (encoding human mitochondrial pre-tRNA^Asn^ and pre-tRNA^Ala^) as templates. All PCR primers and templates are listed in Supplementary Table S2.

The templates for the *in vitro* transcription of *Tt* pre-tRNA^Gly^ with human mitochondrial leader sequences from pre-tRNA^Ala^ or pre-tRNA^Tyr^, and for variants G_−1_-C_73_, U_32_→A_32_, U_33_→A_33_, G_34_→U_34_, A_38_→C_38_ and A_-1_/A_44_/A_45_/A_73_ were generated by overlap-extension PCR using plasmids pSBpt3’hh A_−1_/A_73_ (encoding *Tt* pre-tRNA^Gly^ with A_−1_/A_73_) [32], phA1, and phY1 (encoding human mitochondrial pre-tRNA^Tyr^ with flanking sequences) [7] as templates. Plasmid phY1 was also subjected to overlap-extension PCR to generate templates for transcription of pre-tRNA^Tyr^ with shorter leader lengths, with or without trailer, or with the 5′ leader of *Tt* pre-tRNA^Gly^. PCR primers and templates are listed in Supplementary Table S2.

The *Tt* pre-tRNA^Gly^ variants Aa_b9_T and ΔD were generated by PCR using the previously described plasmid or PCR product as templates [32] and the primers listed in Supplementary Table S2. The templates for the *in vitro* transcription of the combined precursor of human mitochondrial tRNA^Ser(AGY)^ and tRNA^Leu(CUN)^, and the size markers composed of human mitochondrial tRNA^His^ or tRNA^His^ plus tRNA^Ser(AGY)^ were generated by PCR using the primers listed in Supplementary Table S2 and plasmid phHSL-N5 [34] as template.

All aforementioned pre-tRNA substrates were under the control of a T7 RNA polymerase promoter. *In vitro* transcription, 5′-^32^P-end labeling and gel purification were carried out as previously described [35–37]. The concentration of substrates was calculated based on RiboGreen fluorescence using dilutions of unlabeled pre-tRNA as standards as described recently [15].

The human mt tRNA^Ile^ methyltransferase substrate, its synthesis and labeling were previously described [14].

**Figure 1.**
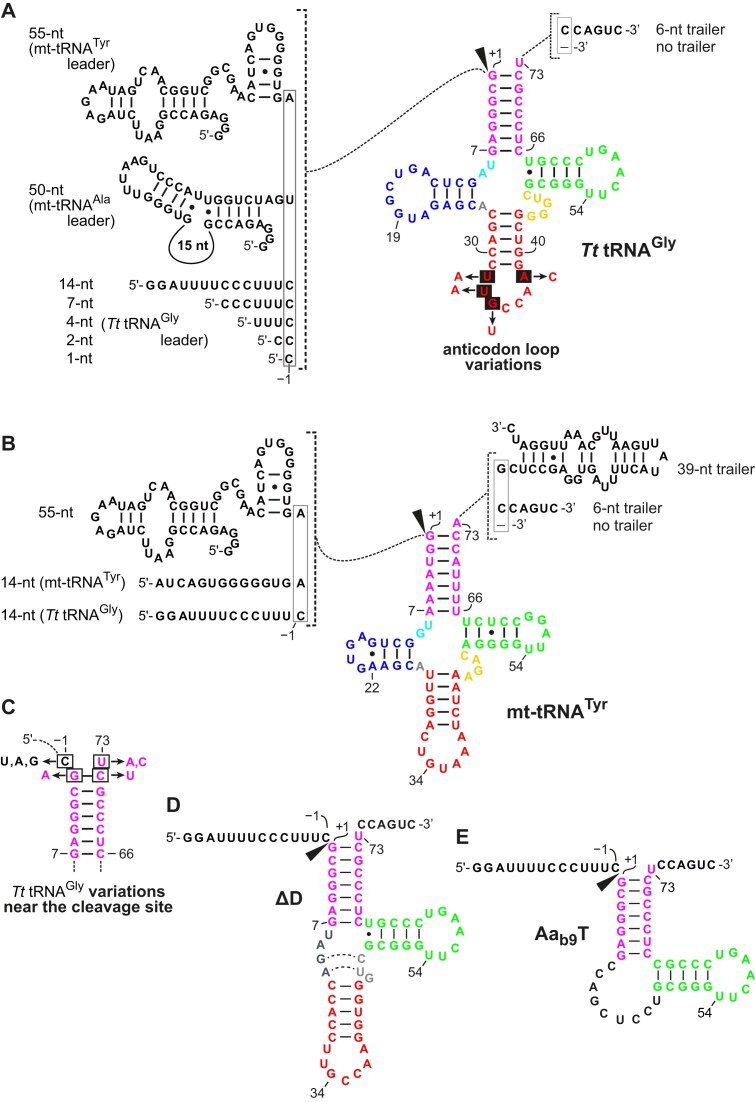
Sequences and predicted secondary structures of analyzed substrates. (**A**) 5′-leader and 3′-trailer variants of *T. thermophilus* (*Tt*) pre-tRNA^Gly^, with the origin of leader sequences indicated on the left. The structural elements of the tRNA body are color-coded: magenta, aminoacyl acceptor stem; blue, D arm; red, AC arm; gold, variable loop; green, T arm. The positions of selected nucleotides are numbered according to convention [59]. The canonical RNase P cleavage site (black arrowhead) is located between nucleotides −1 and +1. Nucleotide exchanges in the AC loop are highlighted by black rectangles in the colored cloverleaf presentation. (**B**) Analyzed 5′-leader and 3′-trailer variants of human mitochondrial tRNA^Tyr^. (**C**) Nucleotide exchanges near the RNase P cleavage site analyzed in the context of *Tt* pre-tRNA^Gly^. (**D**) Truncated *Tt* pre-tRNA^Gly^ variant lacking the D arm; the dashed lines indicate potential base pairings extending the AC stem. (**E**) *Tt* pre-tRNA^Gly^ derivative substrate only consisting of acceptor stem and T arm separated on the 5′ side by a bulge of 9 nt (b9) to allow adaptability of the coaxial stack of acceptor and T stem upon binding to the enzyme.

The sequences and (predicted) secondary structures of all substrates used in this study can be found in either Fig. 1 or Supplementary Fig. S1.

### RNase P cleavage assays

All enzymatic assays and single-turnover kinetics with mtRNase P were performed at 30°C essentially as recently described [15]. The reaction buffer was composed of 50 mM Tris–HCl, pH 7.4, 4.5 mM MgCl_2_, 20 μg/ml bovine serum albumine (BSA), 2 mM dithiothreitol (DTT), and 0.5 units/μg murine RNase inhibitor; a buffer variation with Tris–HCl pH 8.0 was used for the experiments with poorly cleaved substrates (Table 4 and Fig. 3) and less active mutant enzymes (Fig. 7 and Table 6) to accelerate the reaction rate (as previously also demonstrated for *At*PRORP1 [38]). The final pre-tRNA substrate concentration was 0.2 nM. All substrates were subjected to the following denaturation-refolding procedure: the labeled pre-tRNAs were preincubated in reaction buffer at 85°C for 5 min, followed by incubation at room temperature for 10 min and at 37°C for 15 min. For mtRNase P holoenzyme reactions, the TRMT10C–SDR5C1 complex was then added to the refolded substrate. Substrate and enzyme pre-mixes, the latter containing PRORP alone in reaction buffer, were separately incubated at 30°C for 5 min, and the reactions were initiated by combining equal volumes of the two. Reaction aliquots were withdrawn at different time points and processed as previously described [32].

The pseudo first-order rate constant of cleavage (*k*_obs_) was determined through nonlinear regression analysis (Prism, GraphPad Software; or Grafit, Erithacus Software), fitting the data to a single exponential equation: *f*_cleaved_ = *f*_endpoint_ × (1 − e^−(^*^k^*^obs) ×^  *^t^*), where *f*_cleaved_ = fraction of substrate cleaved, *t* = time, *f*_endpoint_ = fraction of maximum cleavable substrate. Alternatively, for reactions proceeding too slow, which did not reach meaningful endpoints within reasonable incubation times, the initial, largely linear phase of the reaction was subjected to linear regression analysis to derive the pseudo first-order rate constant (*k*_obs*_) of cleavage [15]. The maximal rate constant (*k*_react_) and the enzyme concentration at which the half-maximal rate constant is achieved (*K*_M(sto)_) were determined on the basis of *k*_obs_ or *k*_obs*_ values obtained at five or more enzyme concentrations, distributed below and above *K*_M(sto)_, and based on at least three replicate experiments each; *k*_obs_ (or *k*_obs*_) values were plotted against the enzyme concentration, and *k*_react_ and *K*_M(sto)_ were calculated by nonlinear regression analysis (Prism, GraphPad Software; or Grafit, Erithacus Software), fitting the data either to a “Michaelis–Menten-like” enzyme kinetics model, *k*_obs_ = *k*_react_ × [PRORP]/(*K*_M(sto)_ + [PRORP]), or to an allosteric enzyme kinetics model (Hill equation), *k*_obs_ = *k*_max_ × [TRMT10C–SDR5C1]^*n*^/(*K* + [TRMT10C–SDR5C1]^*n*^), with *n* representing the Hill coefficient.

Single-turnover kinetics with *At*PRORP3 were carried out at 21°C in 50 mM Tris–HCl pH 7.1, 4.5 MgCl_2_, 20 μg/ml BSA, 2 mM DTT, and 0.5 units/μg murine RNase inhibitor. Otherwise, experimental procedure and analysis were identical to those described above for mtRNase P.

### Methyltransferase assay of TRMT10C and its variants

Methylation was carried out under single-turnover conditions in 50 mM Tris–HCl pH 8, 4.5 MgCl_2_, 20 μg/ml BSA, 2 mM DTT, 0.5 units/μg murine RNase inhibitor, and 25 µM *S*-adenosyl methionine (SAM). Human mitochondrial pre-tRNA^Ile^ (Supplementary Fig. S1) internally ^32^P-labeled 5′ of position 9 [14] at a concentration of 1 nM was incubated in processing buffer as described above. Native TRMT10C and all recombinant proteins, in complex with SDR5C1, were used at a final concentration of 200 nM. Substrate and enzyme solutions were preincubated separately at 30°C for 5 min. Reactions (at 30°C) were started by combining equal volumes of the two solutions. Aliquots were withdrawn at specific time points and processed as previously described [14]. The pseudo first-order rate constants *k*_obs_ or *k*_obs*_ were determined as described above for mtRNase P.

## Results

### The role of 5′ and 3′ flanking sequences in pre-tRNA processing by human mtRNase P

In bacterial RNase P, the immediate 5′- and 3′-flanking sequences of the tRNA play a crucial role in substrate recognition and cleavage-site selection [29, 39]. However, in the case of the nuclear single-polypeptide PRORP of *A. thaliana* (*At*PRORP3) neither the presence of 5′-leader nor 3′-trailer sequences including the 3′-terminal CCA affected pre-tRNA processing to a significant extent [32]. The role of tRNA extensions has so far not been studied for human mtRNase P. Noteworthy, human mitochondrial tRNAs are transcribed as part of long polycistronic units; some are flanked by full-length mRNAs or rRNAs, others are separated from neighboring tRNAs by a few nucleotides, are butt-jointed, or even overlap by 1 nt [40–44]. Thus, human mtRNase P has to cope with a large variety of tRNA 5′- and 3′-environments.

We first tested variants of the *T. thermophilus* (*Tt*) pre-tRNA^Gly^ model substrate with its 5′-leader progressively shortened from 14 to 1 nt (Fig. 1A). In addition, we compared *Tt* pre-tRNA^Gly^ carrying a 6-nt long 3′-trailer with a variant lacking this trailer (3′-end at the discriminator U_73_). We used this pre-tRNA^Gly^ substrate because it was analyzed in a corresponding study on *At*PRORP3 [32] and is processed *in vitro* by human mtRNase P as well as by human PRORP alone [15]. Shortening the leader length to 7 nt or deletion of the trailer had essentially no effect on the processing by human mtRNase P, while further reduction of the leader to 4 and 2 nt resulted in minor gradual reductions of cleavage rate (Table 1). However, the precursor with a 1-nt leader was cleaved substantially less efficient (∼5-fold reduced *k*_obs_) compared to the parental *Tt* pre-tRNA^Gly^ with the 14-nt leader. A detailed kinetic analysis confirmed the lower efficiency of the 1-nt versus 14-nt leader substrate (2- to 3-fold decrease in *k*_react_ and an ∼9-fold increase in *K*_M(sto)_; Table 1). Despite the difference in cleavage efficiency between the 1-nt leader variant and the other variants with longer leaders, all tested substrates reached a similar extent of cleavage (>93%) after 60 min of incubation. This excludes the possibility that the 1-nt leader might give rise to nonproductive binding modes to the enzyme.

**Table 1.** Role of 5′-leader length variation in pre-tRNA processing by human mtRNase P

	*k*_obs_ (min^−1^)^a^	*k*_react_ (min^−1^)^b^	*K*_M(sto)_ (nM)^b^
**14-nt leader** ^c^	1.61 ± 0.04	1.91 ± 0.10	77 ± 11
**7-nt leader**	1.72 ± 0.14		
**4-nt leader**	1.44 ± 0.13		
**2-nt leader** ^d^	1.18 ± 0.09		
**1-nt leader**	0.34 ± 0.02	0.71 ± 0.09	669 ± 169
**14-nt leader, no 3′-trailer** ^e^	1.55 ± 0.08		

Single-turnover rate constants for the processing of *Tt* pre-tRNA^Gly^ with different 5′-leader lengths by PRORP in the presence of TRMT10C–SDR5C1.

^a^ *k*_obs_ was determined at 500 nM PRORP and 600 nM TRMT10C–SDR5C1 (mean ± standard deviation derived from fitting the data to the equation for a single exponential, based on 6 ± 1 replicate experiments each).

^b^The maximal rate constant (*k*_react_) and the enzyme concentration at which the half-maximal rate constant is achieved (*K*_M(sto)_) were determined by a Michaelis–Menten-like kinetics model, utilizing trace amounts of substrate and increasing concentrations of PRORP at a constant concentration of 600 nM TRMT10C–SDR5C1 (best-fit values ± standard error based on 4 to 5 replicate experiments for each PRORP concentration).

^c^The parental *Tt* pre-tRNA^Gly^ has a leader of 14 nucleotides and a trailer of 6 nucleotides (U_73_-CCAGUC-3′ including the CCA sequence; see Fig. 1).

^d^The sequence of the leader is 5′-CC and therefore differs from the native leader sequence at position −2.

^e^The aminoacyl acceptor stem of this *Tt* pre-tRNA^Gly^ variant comprises only of the discriminator nucleotide U_73_ at the 3′-end.

We also tested 5′-leader length variants of the natural human mtRNase P substrate pre-tRNA^Ala^ (Supplementary Fig. S1A). Shortening the 50-nt leader of the previously characterized substrate [15] to 7, 2, or 1 nt again resulted in a reduction of the cleavage rate (Supplementary Table S3). Detailed kinetic analysis revealed an ∼5-fold reduced *k*_react_ value for the pre-tRNA^Ala^ with a 1-nt leader compared to the substrate with the 50-nt long leader, while *K*_M(sto)_ dropped ∼2-fold (Supplementary Table S3 and Supplementary Fig. S2) rather than increasing as observed for *Tt* pre*-*tRNA^Gly^ upon leader shortening from 14 to 1 nt (Table 1). Also, the ∼2.5-fold drop in *k*_obs_ for a pre-tRNA^Ala^ with a 7-nt versus 50-nt leader appears remarkable, indicating that leaders almost as long as the tRNA itself may accelerate cleavage kinetics. However, further increasing the length of the leader to include the complete mitochondrial tRNA^Asn^ preceding tRNA^Ala^ in the mitochondrial genome (Supplementary Fig. S1), moderately reduced the cleavage rate (Supplementary Table S3; 74-nt leader), probably because of the additional tRNA structure competing for mtRNase P binding.

We validated the impact of considerably longer 5′-leaders (50 or 55 nt) in the context of the bacterial tRNA^Gly^ (Fig. 1A) and human mitochondrial tRNA^Tyr^ (Fig. 1B). Here, we used a lower concentration of PRORP (20 nM) because tRNA^Tyr^ flanked by the 55-nt leader and 39-nt trailer was cleaved at a rate of >10 min^-1^ at 500 nM PRORP/600 nM TRMT10C–SDR5C1 [15], which precluded reliable kinetics measurements by manual sampling. In addition, we expected that conditions of subsaturating PRORP concentrations are more sensitive to leader/trailer effects on substrate binding. *Tt* pre-tRNA^Gly^ equipped with a 6-nt 3′-trailer and the 50-nt leader derived from mitochondrial pre-tRNA^Ala^ was cleaved 1.4-fold faster by mtRNase P than the corresponding *Tt* pre-tRNA^Gly^ variant with the 14-nt leader, and the same tRNA carrying the 55-nt leader derived from mitochondrial pre-tRNA^Tyr^ was cleaved even 2.4-fold faster (Table 2, left part). These observations consistently indicate that extension of the 5′-leader does not impair, but even moderately enhances the efficiency of pre-tRNA processing by mtRNase P. We observed a similar trend when testing the same *Tt* pre-tRNA^Gly^ leader variants but in the context of a trailerless tRNA. Noteworthy, the absence of the trailer caused slight general increases in processing efficiency (1.3- to 1.8-fold) relative to the corresponding substrates with the 6-nt 3′-trailer (Table 2, left part). We further generated variants of human mitochondrial tRNA^Tyr^ with its 55-nt leader shortened to 14 nt, or equipped with the 14-nt leader of *Tt* pre*-*tRNA^Gly^, either combined with the native 39-nt trailer, a short 6-nt trailer or without trailer (ending with the discriminator nt 73). Combining the mitochondrial tRNA^Tyr^ with the 55-nt leader increased *k*_obs_ ∼5-fold (6-nt trailer) and >2-fold (no trailer) relative to the same tRNA body equipped with either of the two 14-nt leaders (Table 2, right part). Also, tRNA^Tyr^ with the 55-nt leader was cleaved 3.3-fold (6-nt trailer) or 2.4-fold (no trailer) faster than the *Tt* pre*-*tRNA^Gly^ carrying the same 5′ and 3′ extensions (2.6 versus 0.8 and 2.5 versus 1.1 min^−1^, Table 2), while tRNA^Tyr^ with the 14-nt leader of *Tt* pre*-*tRNA^Gly^ was cleaved only 1.5-fold (6-nt trailer) or 1.9-fold (no trailer) faster than the corresponding substrate with the tRNA^Gly^ body (0.5 versus 0.3 and 1.1 versus 0.6 min^−1^). This points to synergistic effects of the 55-nt leader and the tRNA^Tyr^ body that go beyond the effect of the 55-nt leader when combined with *Tt* tRNA^Gly^. As observed for the *Tt* pre*-*tRNA^Gly^ variants, the absence of any trailer nucleotides enhanced cleavage of the 14-nt leader variants of tRNA^Tyr^ (Table 2, right part). Likewise, the 55-nt leader variant of tRNA^Tyr^ with the 39-nt trailer was cleaved ∼1.6-fold slower than the 6-nt trailer variant or the trailerless variant. Thus, we observed a trend toward faster cleavage when substrates were equipped with a short or no 3′-trailer.

**Table 2.** Role of 5′-leader and 3′-trailer length variations in processing of bacterial *Tt* pre-tRNA^Gly^ and human mitochondrial pre-tRNA^Tyr^ by human mtRNase P

	*Tt* pre-tRNA^Gly^			human mitochondrial pre-tRNA^Tyr^		
	6-nt trailer^a^	no trailer^b^		39-nt trailer^c^	6-nt trailer^a^	no trailer^b^
	*k*_obs_ (min^−1^)	*k*_obs_ (min^−1^)		*k*_obs_ (min^−1^)	*k*_obs_ (min^−1^)	*k*_obs_ (min^−1^)
14-nt leader of *Tt* pre-tRNA^Gly^	0.33 ± 0.02	0.60 ± 0.06	14-nt leader of *Tt* pre-tRNA^Gly^		0.48 ± 0.03	1.13 ± 0.09
50-nt leader of pre-tRNA^Ala^	0.47 ± 0.02	0.81 ± 0.004	14-nt leader of pre-tRNA^Tyr^	0.89 ± 0.07	0.58 ± 0.04	1.05 ± 0.07
55-nt leader of pre-tRNA^Tyr^	0.79 ± 0.04	1.05 ± 0.02	55-nt leader of pre-tRNA^Tyr^	1.61 ± 0.08	2.59 ± 0.15	2.48 ± 0.20

Single-turnover rate constants (*k*_obs_) for the processing of *Tt* pre-tRNA^Gly^ and mitochondrial pre-tRNA^Tyr^ by human PRORP in the presence of TRMT10C–SDR5C1 as a function of leader and trailer length, determined at 20 nM PRORP and 600 nM TRMT10C–SDR5C1 (mean ± standard deviation derived from fitting the data to the equation for a single exponential, based on 4 to 9 replicate experiments each).

^a^The aminoacyl acceptor stem comprises a trailer of 6 nucleotides (U_73_-CCAGUC-3′ including the CCA sequence; see Fig. 1).

^b^The aminoacyl acceptor stem carries only the discriminator nucleotide (U_73_) at its 3′ end.

^c^The aminoacyl acceptor stem carries parts of the mitochondrial tRNA^Cys^ sequence at its 3′ end.

Finally, we investigated the effect of the above-described leader extensions on pre-tRNA processing by *At*PRORP3. This expands our previous study on substrate recognition and cleavage-site selection by *At*PRORP3 where we only analyzed substrates with leaders ≤14 nt [32]. The analysis was confined to variants of *Tt* pre-tRNA^Gly^ because human mitochondrial pre-tRNA^Tyr^ is not processed by *At*PRORP3. In the reactions catalyzed by *At*PRORP3, combinations of the different leader lengths and the presence or absence of the 6-nt trailer resulted in less coherent changes, with the highest *k*_obs_ value measured for the combination of the 50-nt leader and 6-nt trailer (Supplementary Table S4). In the reaction with human mtRNase P, *Tt* pre-tRNA^Gly^ with the 55-nt leader was cleaved 1.7-fold faster than the one with the 50-nt leader (both with 6-nt trailer, Table 2, left part), whereas this relation was reversed to 2.4-fold slower with *At*PRORP3 (Supplementary Table S4). In contrast to its effect on mtRNase P, deletion of the trailer had no consistent effect on the processing of bacterial pre-tRNA^Gly^ by *At*PRORP3. The observed differential effects of leader and trailer extensions on cleavage efficiency by *At*PRORP3 versus human mtRNase P might reflect the differences in enzyme complexity and substrate recognition by single-subunit PRORP3 versus multiple-subunit mtRNase P.

In summary, our results indicate that processing efficiency by human mtRNase P gradually declines for pre-tRNAs with leaders shortened to 2 nt, to then more strongly drop for substrates with a 1-nt leader (Table 1 and Supplementary Table S3). Interestingly, longer leader sequences do not interfere with the processing of human mtRNase P, but instead can enhance cleavage efficiency, as exemplified by the 55-nt leader of the human mitochondrial tRNA^Tyr^ substrate. The trailer, on the other hand, tends to have a slightly negative effect on processing by mtRNase P.

### Nucleotide identities and base pairing at the cleavage site

Studies on bacterial RNA-based RNase P and *At*PRORP3 highlighted the differential importance of nucleotide identities at the cleavage site. For example, altering the G_1_-C_72_ base pair to U_1_-A_72_, was found to have a negative effect on both binding and catalysis by bacterial RNase P [45, 46], but no significant impact on cleavage by *At*PRORP3 [32]. Furthermore, a U at −1, positioned upstream of the canonical cleavage site, is preferred and conserved in many bacteria and archaea because of its interaction with the RNA subunit of RNase P [47, 48]. In contrast, an A residue at position −1 was found to be favorable for the catalysis by *At*PRORP3 [32]. To investigate the role of nucleotide identity at position −1 in the processing reaction catalyzed by human mtRNase P, we substituted the C_−1_ residue of *Tt* pre*-*tRNA^Gly^ and the U_−1_ of mitochondrial pre-tRNA^Ala^ with the other three nucleotides, respectively (illustrated in Fig. 1C and Supplementary Fig. S1B). For the *Tt* pre*-*tRNA^Gly^ variants with a purine at this position, we changed the identity of the discriminator base from U_73_ to A_73_ to avoid base pairing with N_−1_. For *Tt* pre*-*tRNA^Gly^, the substitution of C_−1_ with U_−1_ or G_−1_ slightly increased the *k*_obs_ (∼1.5-fold), and an A at position −1 caused a 2.2-fold increase in *k*_obs_ and 2.4-fold increase in *k*_react_ (Table 3); altering the identity of the discriminator base alone (variant A_73_) barely affected the rate of cleavage. In the case of mitochondrial pre-tRNA^Ala^, substrates with U_−1_ and C_−1_ were cleaved with essentially identical *k*_obs_ values, while again the relative highest *k*_obs_ was obtained with an A at −1 (∼1.5-fold compared to U_−1_ or C_−1_; Supplementary Table S5). Thus, nucleotide identity at position −1 plays a moderate role in the reaction catalyzed by human mtRNase P and an A residue immediately upstream of the canonical cleavage site is favorable for catalysis, as observed for *At*PRORP3 [32].

**Table 3.** The effect of varying base identity at the cleavage site

	*k*_obs_ (min^−1^)^a^	*k*_react_ (min^−1^)^b^	*K*_M(sto)_ (nM)^b^
***Tt* pre-tRNA^Gly^ (C_-1_, U_73_, G_1_, C_72_**)	1.57 ± 0.04	1.91 ± 0.10	77 ± 11
**U_−1_**	2.59 ± 0.15		
**G_−1_, A_73_** ^c^	2.19 ± 0.09		
**A_−1_, A_73_** ^c^	3.42 ± 0.21	4.58 ± 0.27	187 ± 29
**A_73_**	1.70 ± 0.08		
**U_1_, A_72_**	1.15 ± 0.07		
**G_−1_, C_73_**	1.23 ± 0.04		

Single-turnover kinetic constants for processing of *Tt* pre-tRNA^Gly^ variants (14-nt leader, 6-nt trailer) with different nucleotide identities at positions −1, +73, +1, and/or +72 by human mitochondrial PRORP in the presence of TRMT10C–SDR5C1.

^a^ *k*_obs_ was determined at 500 nM PRORP and 600 nM TRMT10C–SDR5C1 (mean ± standard deviation derived from fitting the data to the equation for a single exponential; based on 5 to 7 replicate experiments each).

^b^ *k*_react_ and *K*_M(sto)_, see legend to Table 1 (based on 3 to 5 replicate experiments each).

^c^The identity of U_73_ was changed to A_73_ to prevent base pairing with nucleotide −1.

**Figure 2.**
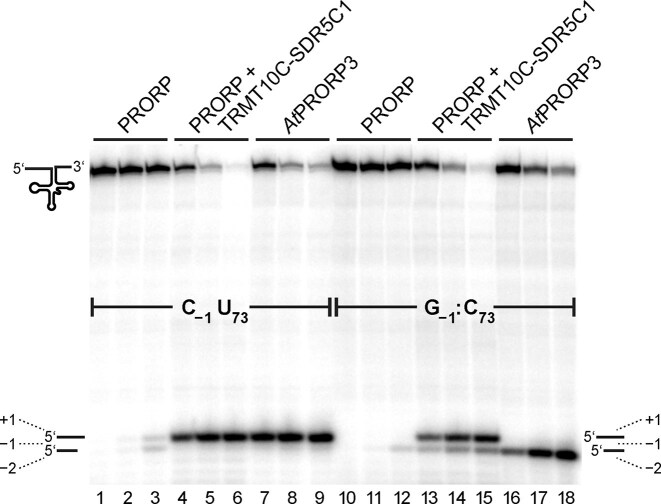
Analysis of cleavage site selection by human PRORP alone (500 nM), the mtRNase P holoenzyme (500 nM PRORP + 600 nM TRMT10C–SDR5C1) and *At*PRORP3 (10 nM) when acting on the parental *Tt* pre-tRNA^Gly^ (with C_-1_, U_73_; lanes 1–9) or the *Tt* pre-tRNA^Gly^ variant with a G_-1_:C_73_ base pair; lanes 10–18). Aliquots were withdrawn from the reactions after 30 s (lanes 1, 4, 7, 10, 13, and 16), 3 min (lanes 2, 5, 8, 11, 14, and 17), and 90 min (lanes 3, 6, 9, 12, 15, and 18), respectively. Reaction products were resolved by 12% denaturing PAGE.

Changing the first base pair next to the cleavage site in *Tt* pre*-*tRNA^Gly^ from G_1_-C_72_ to U_1_-A_72_ caused an ∼1.4-fold decrease in *k*_obs_ relative to the parental substrate (1.2 versus 1.6 min^−1^; Table 3) and also resulted in the lowest experimental endpoint (∼85% ± 3%) relative to the other analyzed variants, all with endpoints above 90%. This suggests an increase in substrate conformers that cannot form productive enzyme–substrate complexes (see also Supplementary Fig. S3). Adding an extra G_−1_-C_73_ base pair to the end of the acceptor stem of *Tt* pre*-*tRNA^Gly^ reduced the overall rate of cleavage by human mtRNase P only marginally (1.3-fold; Table 3), similar to *At*PRORP3 (1.2-fold) [32]. Changing the upstream U_−1_-A_73_ base pair of mitochondrial pre-tRNA^Ala^ to the stronger G_−1_-C_73_ base pair reduced *k*_react_ only little more (1.6-fold; Supplementary Table S5 and Supplementary Fig. S4) than observed in the context of *Tt* pre*-*tRNA^Gly^. However, while an extra G-C base pair shifted cleavage by *At*PRORP3 completely to 1 nt upstream between positions −1 and −2 (Fig. 2, compare lanes 7–9 and 16–18) (see also [32]), human mtRNase P still cleaved ~75% of the *Tt* pre*-*tRNA^Gly^ and the mitochondrial pre-tRNA^Ala^ molecules at the canonical cleavage site (compare Fig. 2, lanes 4–6 to 13–15, and Supplementary Fig. S5, lanes 6–8 to 12–14, respectively). Intriguingly, human PRORP alone behaved more like *At*PRORP3 on the G_−1_-C_73_ substrates, cleaving exclusively between −1 and −2 in the case of *Tt* pre*-*tRNA^Gly^ (Fig. 2, lane 12); for mitochondrial pre-tRNA^Ala^, cleavage by PRORP alone also shifted toward the upstream cleavage site, but this effect was less pronounced, resulting in about equal cleavage at the canonical and the upstream site (Supplementary Fig. S5, lane 11).

### Structural domains of the tRNA, truncated pre-tRNA substrates

For plant protein-only forms of RNase P as well as for bacterial, archaeal and eukaryal nuclear RNP forms, the acceptor stem and T domain constitute the major recognition/interaction surface between pre-tRNA substrate and enzyme. These structural elements correspond to the “short arm” and part of the “elbow” of the inverted “L” in the L-shaped tRNA structure, and substrates lacking the D and AC arm (i.e. stem-loop) can be processed by these enzymes with only moderate reductions in cleavage efficiency [32, 49–53]. Here, we investigated whether human PRORP is also capable of processing similarly truncated substrates and whether TRMT10C can stimulate PRORP cleavage in the absence of major interactions between TRMT10C and the pre-tRNA. For this purpose, we tested cleavage of *Tt* tRNA^Gly^ variants with specific domain deletions in the presence of close-to-saturating PRORP (500 nM) and TRMT10C–SDR5C1 (600 nM) concentrations, as well as with human PRORP alone. We removed the D domain (ΔD, Fig. 1D) and we minimized the tRNA structure to consist only of the acceptor stem connected to the T stem-loop through a bulge of 9 nt (Aa_b9_T, Fig. 1E).

Compared with the parental *Tt* pre-tRNA^Gly^, mtRNase P cleaved the ΔD substrate at an ∼70-fold reduced rate and an experimental endpoint of 27 versus 97% (Table 4). For the minimized substrate Aa_b9_T, this deteriorated to an ∼3000-fold decreased rate, with ∼10% of substrate cleaved after 150 min of incubation. These results correlate with the progressive lack of structural tRNA elements that are involved in the interaction with TRMT10C according to the cryo-EM structure of human mtRNase P [22]. The cleavage rate of Aa_b9_T by human PRORP alone was only ∼5-fold decreased compared to that of *Tt* pre-tRNA^Gly^ (Table 4), consistent with PRORP primarily recognizing acceptor stem and T loop. In contrast to Aa_b9_T, no cleavage at all was detectable for substrate ΔD in the PRORP-alone reaction, which may be attributed to a constrained, suboptimal orientation of the T arm relative to the acceptor stem, due to an AC stem extension caused by potential pairings between bases in the variable loop with those connecting acceptor and AC stem (indicated by dashed lines in Fig. 1D). Substrate Aa_b9_T was cleaved with equally low efficiency by PRORP alone and by the holoenzyme (7 × 10^–4^ min^−1^; Table 4), indicating that Aa_b9_T binds exclusively to PRORP, but not to TRMT10C–SDR5C1. Taken together our results demonstrate that the mtRNase P holoenzyme acts inefficiently on truncated tRNAs lacking the D arm and even less on tRNA derivatives lacking both, the D and the AC arm. These findings substantiate that all subdomains of mitochondrial tRNAs contribute to substrate incorporation into the holoenzyme complex. Nevertheless, the ancient PRORP-binding mode appears sufficiently conserved to enable human PRORP alone to process a minimal substrate comprising acceptor stem and T arm only, although with very low efficiency.

**Table 4.** Cleavage of truncated and minimized substrates, and of tRNA^Leu(CUN)^ and tRNA^Ser(AGY)^ from their natural context

	mtRNase P*k*_obs_ or *k*_obs*_ (min^−1^)	Endpoint (%)		PRORP alone*k*_obs*_ (min^−1^)
		Fit	Exp.	
***Tt* pre-tRNA^Gly^**	2.13 ± 0.09	92.9	97.2	(3.4 ± 0.4) x 10^–3^
**ΔD**	(3 ± 0.6) × 10^–2^	27.5	26.7 (at 90 min)	n.d.
**Aa_b9_T**	(7 ± 0.3) × 10^–4^	–		(7 ± 0.9) x 10^–4^
**tRNA^Leu(CUN)^** ^a^	0.5 ± 0.03 (1st)			
	0.15 ± 0.05 (2nd)			
**tRNA^Ser(AGY)^**	(3 ± 0.1) × 10^–3^			

Single-turnover rate constants (*k*_obs_ or *k*_obs__*_) for the processing of *Tt* pre-tRNA^Gly^ variants (14-nt leader, 6-nt trailer) lacking the D arm (ΔD) or composed of the aminoacyl acceptor stem (Aa) and TψC domain (T) only (Aa_b9_T, see Fig. 1), and of mitochondrial tRNA^Leu(CUN)^ and tRNA^Ser(AGY)^ by human PRORP (500 nM) in the presence or absence of TRMT10C-SDR5C1 (600 nM). Given are mean values ± standard deviation derived from fitting the data to the equation for a single exponential (*k*_obs_) for cleavage of *Tt* pre-tRNA^Gly^, ΔD, and tRNA^Leu(CUN)^ by mtRNase P, or by linear fits (*k*_obs*_) in all other cases (based on at least three replicate experiments each). The endpoint of the reaction is either the endpoint calculated by curve fitting (fit) or the extent of cleavage determined experimentally (exp.) at the final indicated time point. n.d., not detectable.

^a^The first value is the initial rapid burst derived from a linear approximation of the first phase, the second value derived from fitting the later data points to the equation for a single exponential.

One of the two human mitochondrial tRNA^Ser^ isoacceptors, tRNA^Ser(AGY)^, resembles the artificial ΔD pre-tRNA substrate. In the mitochondrial genome, this D armless tRNA^Ser(AGY)^ is embedded between the tRNA^His^ and tRNA^Leu(CUN)^ genes (Supplementary Fig. S1C). The precise flanking of tRNA^Ser(AGY)^ at its 5′ end by tRNA^His^ was many years ago shown to be crucial for an indirect 5′-maturation mechanism by RNase Z processing the 3′ end of tRNA^His^ and thereby concomitantly generating the mature 5′ end of tRNA^Ser(AGY)^ [34]. Crude mitochondrial extracts containing all processing enzymes had been employed in these experiments and, given the result with the ΔD pre-tRNA substrate, we decided to (re-)examine the 5′-end processing of tRNA^Ser(AGY)^ by recombinant mtRNase P within its natural context (i.e. embedded between tRNA^His^ and tRNA^Leu(CUN)^) and in the absence of RNase Z.

**Figure 3.**
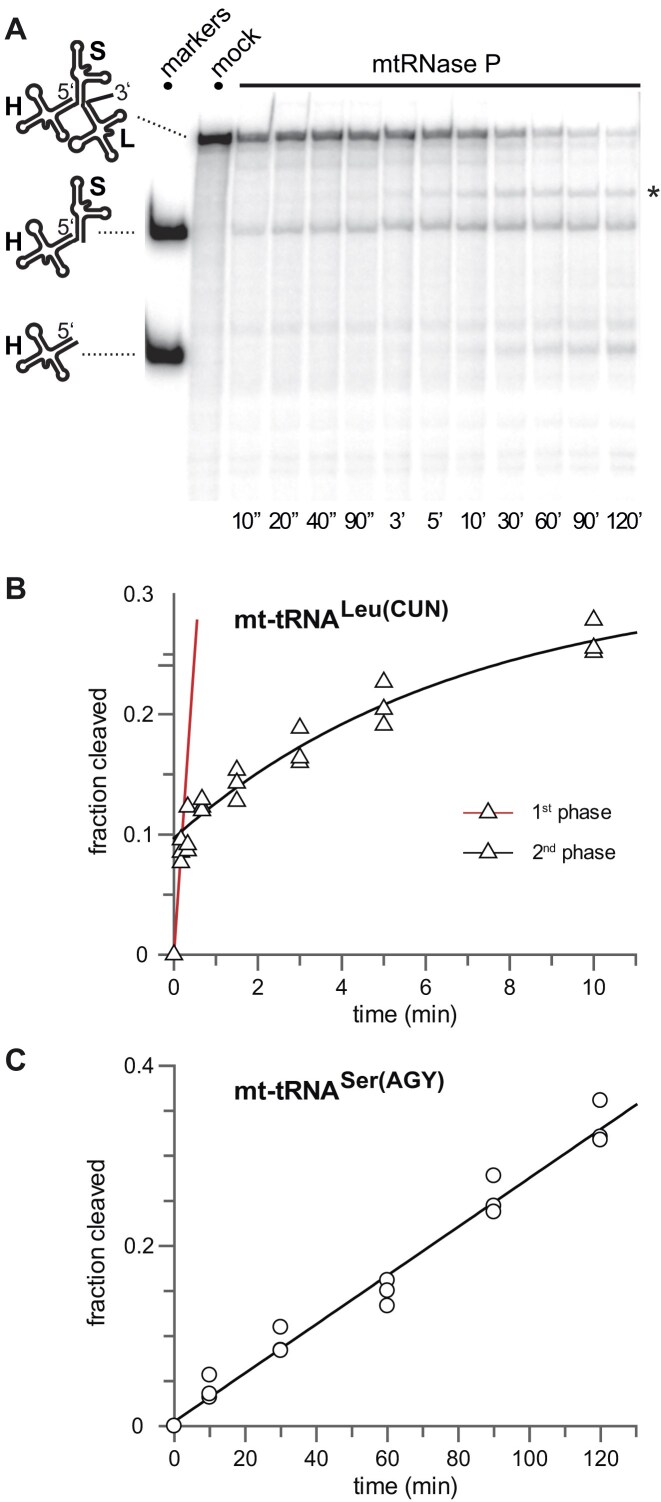
mtRNase P processing kinetics of a T7 transcript (199 nt) corresponding to the section of the human mitochondrial genome encoding the three consecutive mitochondrial tRNAs, tRNA^His^ (H), tRNA^Ser(AGY)^ (S), and tRNA^Leu(CUN)^ (L); the transcript starts with the 5′-terminal nt of tRNA^His^, and terminates 33 nt downstream of tRNA^Leu(CUN)^ in the flanking ND5 gene (Supplementary Fig. S1); mtRNase P was reconstituted from 500 nM PRORP and 600 nM TRMT10C-SDR5C1. (**A**) Representative phosphorimage of a 10% denaturing PAGE analysis of mtRNase P processing kinetics. Lane “markers”: 5′-^32^P-endlabeled RNA fragments corresponding to the 5′-product released after 5′-end maturation of tRNA^Leu(CUN)^ (upper band) and tRNA^His^ only resulting from 5′-end maturation of tRNA^Ser(AGY)^, as sketched on the left. Lane “mock”: Incubation of substrate without enzyme for 120 min. For all other lanes the time of incubation with mtRNase P is indicated at the bottom of each lane. The asterisk to the right labels a by-product resulting from cleavage within tRNA^Leu(CUN)^ for which the exact position has not been determined. (**B**) Kinetics of mtRNase P-catalyzed 5′-end maturation of tRNA^Leu(CUN)^, characterized by an initial burst phase (first phase, red line) and a slower second reaction phase (black curve). (**C**) Time course of tRNA^His^ accumulation as a readout for mtRNase P-catalyzed 5′-end maturation of tRNA^Ser(AGY)^, best described by a linear relationship. For further details, see Table 4.

From a precursor composed of the three aforementioned tRNAs with two potential RNase P cleavage sites (Supplementary Fig. S1C), the 5′ end of tRNA^Leu(CUN)^ was cleaved in an initial rapid burst with a subsequent slower phase following first order kinetics (Fig. 3A and B, and Table 4). Cleavage at the 5′ end of tRNA^Ser(AGY)^ was detected only much later, not earlier than 10 min after the start of the reaction, and progressed with very slow, linear kinetics (Fig. 3A and C, and Table 4). The cleavage kinetics of this substrate mimicking the natural mitochondrial primary transcript are in line with that of the artificial ΔD pre-tRNA and consistent with the previously proposed pathway, whereby the 5′-end maturation of tRNA^Ser(AGY)^ occurs primarily via 3′-end processing of tRNA^His^ by RNase Z [34].

### Impact of the interactions between TRMT10C–SDR5C1 and the anticodon loop on cleavage by human mtRNase P

**Figure 4.**
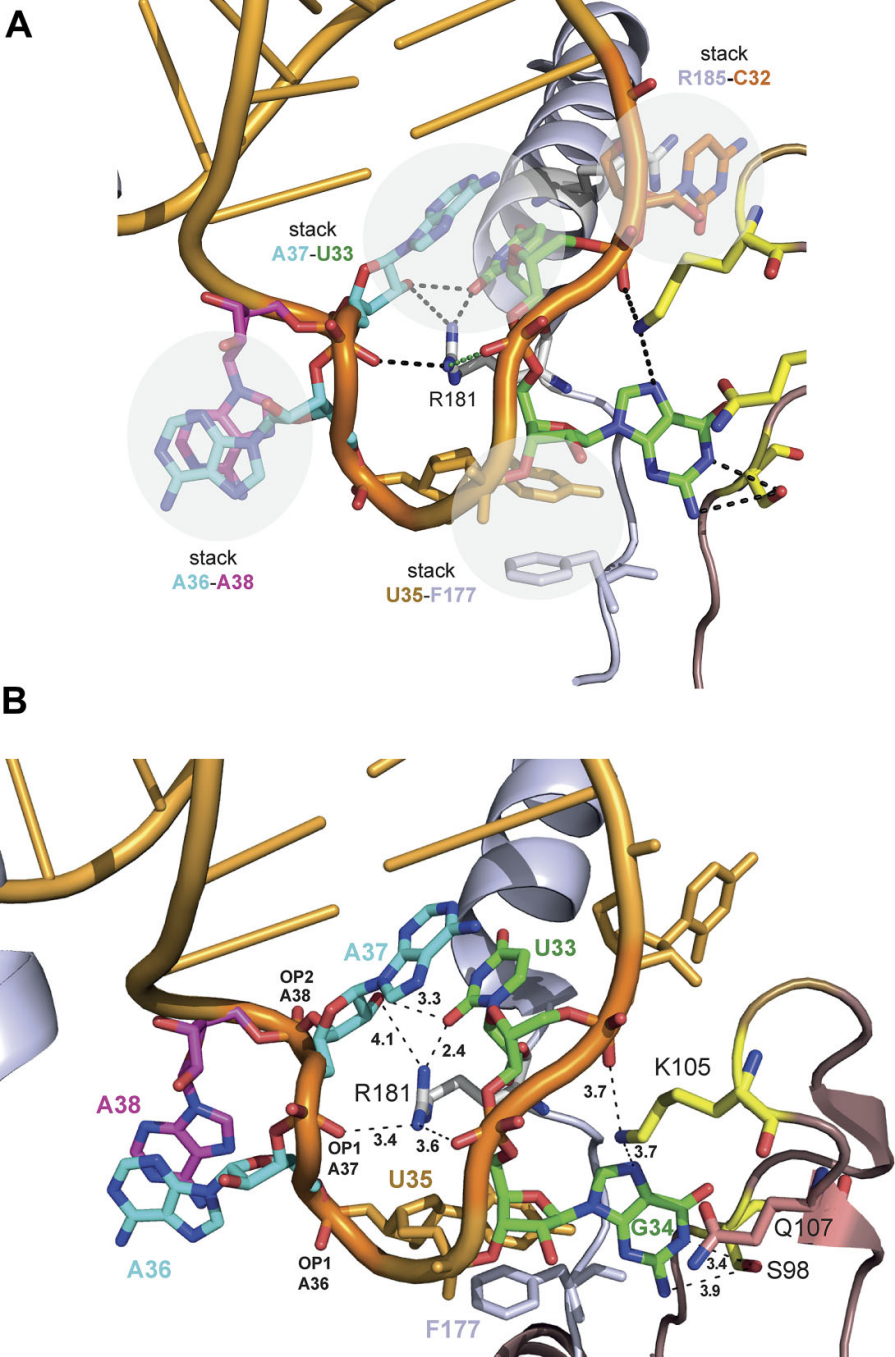
AC interactions of the TRMT10C–SDR5C1 subcomplex in the cryo-EM structure of human mtRNase P in complex with a human mitochondrial pre-tRNA^Tyr^ (PDB: 7ONU) [22]. (**A**) Illustration of observed stacking interactions (gray-shaded spheres) involving AC nucleotides C32, U33, U35, A36, A37, A38, as well as R181, F177, and R185 of TRMT10C that are part of the protein’s adapter loop (aa 175–182) and adapter helix (R185). (**B**) Distances (in Å) of potential H-bond interactions; S98, K105, and Q107 are SDR5C1 residues; a potential interaction of Q107 with AC nucleotide 34 remains speculative based on the cryo-EM structure.

The cryo-EM structure of human mtRNase P revealed extensive interactions of the enzyme complex with the entire tRNA [22]. Particular surprising were the observed interactions between TRMT10C–SDR5C1 and the tRNA’s AC loop (Fig. 4), which are generally not found in other forms of RNase P. One of the suggested interactions was the contact between G_34_ of human mitochondrial tRNA^Tyr^ and SDR5C1 via the backbone and side chains of S98 and K105 (see Fig. 4B). In addition, TRMT10C–SDR5C1 engages in multiple interactions with the AC loop along TRMT10C’s adapter loop and helix, with contacts observed between F177, R181, and R185 of TRMT10C and U_35_, U_33_/A_37_, and C_32_, respectively (illustrated in Fig. 4A and B). To gain mechanistic insight into the role of these interactions, we systematically tested variants of *Tt* pre-tRNA^Gly^ with substitutions G_34_→U_34_, U_32_→A_32_ or U_33_→A_33_; we used *Tt* pre-tRNA^Gly^ variant A_−1_/A_73_ as a basis for these substitutions, because of the higher cleavage rate associated with an A at position −1.

**Figure 5.**
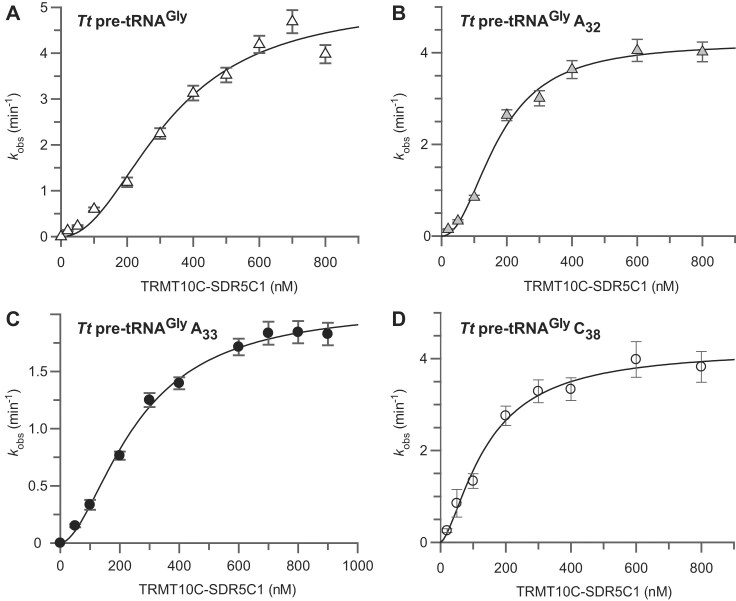
Single-turnover kinetics of the processing of *Tt* pre-tRNA^Gly^ A_−1_/A_73_ (14-nt leader, 6-nt trailer) variants with base exchanges at positions 32, 33, or 38 by human mitochondrial PRORP as a function of the TRMT10C–SDR5C1 concentration. Best fittings were obtained with a version of the Hill equation. Concentrations of TRMT10C–SDR5C1 were varied from 20 to 800 nM at fixed PRORP concentration of 500 nM (best-fit values ± standard error based on the fitting of 4 to 9 replicate experiments each). (**A**) *Tt* pre-tRNA^Gly^ carrying A residues at positions -1 and 73. (**B** and **C**) The same substrate as in panel A but with a U to A exchange at position 32 (B) or 33 (C). (**D**) A substrate like in panel A with A_38_ changed to C_38_ and in addition G to A exchanges at positions 44 and 45 in the variable loop; see legend to Table 5 for further details.

G_34_→U_34_ did not negatively affect cleavage by mtRNase P at low or high PRORP concentrations (20 or 500 nM), suggesting that the SDR5C1 contacts with position 34 are either not G-specific or of minor importance for substrate binding (Table 5). This finding is consistent with the fact that position 34 is part of the AC triplet and consequently not conserved. In contrast, positions 32 and 33 are highly conserved as a pyrimidine and uridine, respectively, in tRNAs in general [26, 54], and strictly as pyrimidines in human mitochondrial tRNAs [28, 55]. Therefore, we hypothesized that purine transversions at these positions could have a more severe impact on processing by human mtRNase P. Here, we varied the concentration of TRMT10C–SDR5C1 from 20 to 800 nM at a fixed PRORP concentration (500 nM) and trace amounts of substrate (0.1 nM). In these experiments we noticed that the plots of *k*_obs_ over TRMT10C–SDR5C1 concentration showed sigmoidal behavior (Fig. 5). Therefore, the TRMT10C–SDR5C1 titration curves were fit to an allosteric Hill equation (Table 5). The substrate variant with the U_33_→A_33_ exchange exhibited a 2.5-fold decrease in *k*_max_ compared to the reference pre-tRNA^Gly^ A_−1_/A_73_, while the *K* value was hardly affected (1.3-fold decreased, Table 5). The U_32_→A_32_ exchange marginally influenced the single turnover kinetics catalyzed by mtRNase P, displaying a similar *k*_max_ as the wild-type tRNA and an ∼2-fold reduced *K* value (Table 5). The beneficial “*K*_m_ effect” may result from preventing potential base pairing between U_32_ and A_38_ in *Tt* pre-tRNA^Gly^ as a consequence of the A_32_ substitution. Such a base pairing would be inconsistent with the AC loop conformation in the cryo-EM structure where N_32_ and N_38_ are in a “flipped out” state (Fig. 4) [22]. In accordance, positions N_32_ and N_38_ cannot form canonical base pairs in the majority of mitochondrial tRNAs. To further examine the significance of potential base pairing between U_32_ and A_38_ for processing by mtRNase P, we generated an A_38_→C_38_ variant while keeping U_32_ (Table 5). The A_38_→C_38_ variant substrate showed a beneficial “*K*_m_ effect” as well, similar to that of the U_32_→A_32_ variant, consistent with the hypothesis that an open AC conformation is favorable for interaction with the mtRNase P holoenzyme.

**Table 5.** The effect of altered base identities in the AC loop

	*k*_obs_ (min^−1^)^a^		
	20 nM PRORP	500 nM PRORP	
***Tt* pre-tRNA^Gly^**	0.42 ± 0.06	4.29 ± 0.38	
**variant G_34_→U_34_**	0.38± 0.03	4.88 ± 0.33	
	***k*_max_** (min^−1^)^b^	***K*** (nM)^b^	**Hill coefficent** ^b^
***Tt* pre-tRNA^Gly^**	5.10 ± 0.62	327 ± 49	2.14 ± 0.48
**variant U_32_→A_32_**	4.22 ± 0.18	174 ± 13	2.17 ± 0.27
**variant U_33_→A_33_**	2.07 ± 0.08	254 ± 16	1.80 ± 0.16
**variant A_38_→C_38_**	4.22 ± 0.27	141 ± 19	1.53 ± 0.23

Single-turnover kinetic constants for processing of *Tt* pre-tRNA^Gly^ A_−1_/A_73_ (14-nt leader, 6-nt trailer) with different base identities at positions 32, 33, 34, and 38 by human mitochondrial PRORP in the presence of TRMT10C–SDR5C1.

^a^ *k*_obs_ was determined at 20 or 500 nM PRORP and 600 nM TRMT10C–SDR5C1 (mean ± standard error).

^b^The best fittings were obtained with a version of the Hill equation. Concentrations of TRMT10C–SDR5C1 were varied from 20 to 800 nM at fixed PRORP concentration of 500 nM (best-fit values ± standard error based on the fitting of 4 to 9 replicate experiments each). The used *Tt* pre-tRNA^Gly^ carried A residues at positions -1 and 73; *Tt* pre-tRNA^Gly^ C_38_ additionally carried G to A exchanges at positions 44 and 45 in the variable loop. Yet, *Tt* pre-tRNA^Gly^ variants A_−1_/G_44_/G_45_/A_73_ and A_−1_/A_44_/A_45_/A_73_ showed indistinguishable kinetics for cleavage by mtRNase P at 500 nM PRORP and 600 nM TRMT10C-SDR5C1 in side-by-side experiments (*k*_obs_ = 3.6 ± 0.4 and 3.6 ± 0.6 min^−1^).

### The role of TRMT10C in the docking of tRNA and the activation of human mtRNase P

**Figure 6.**
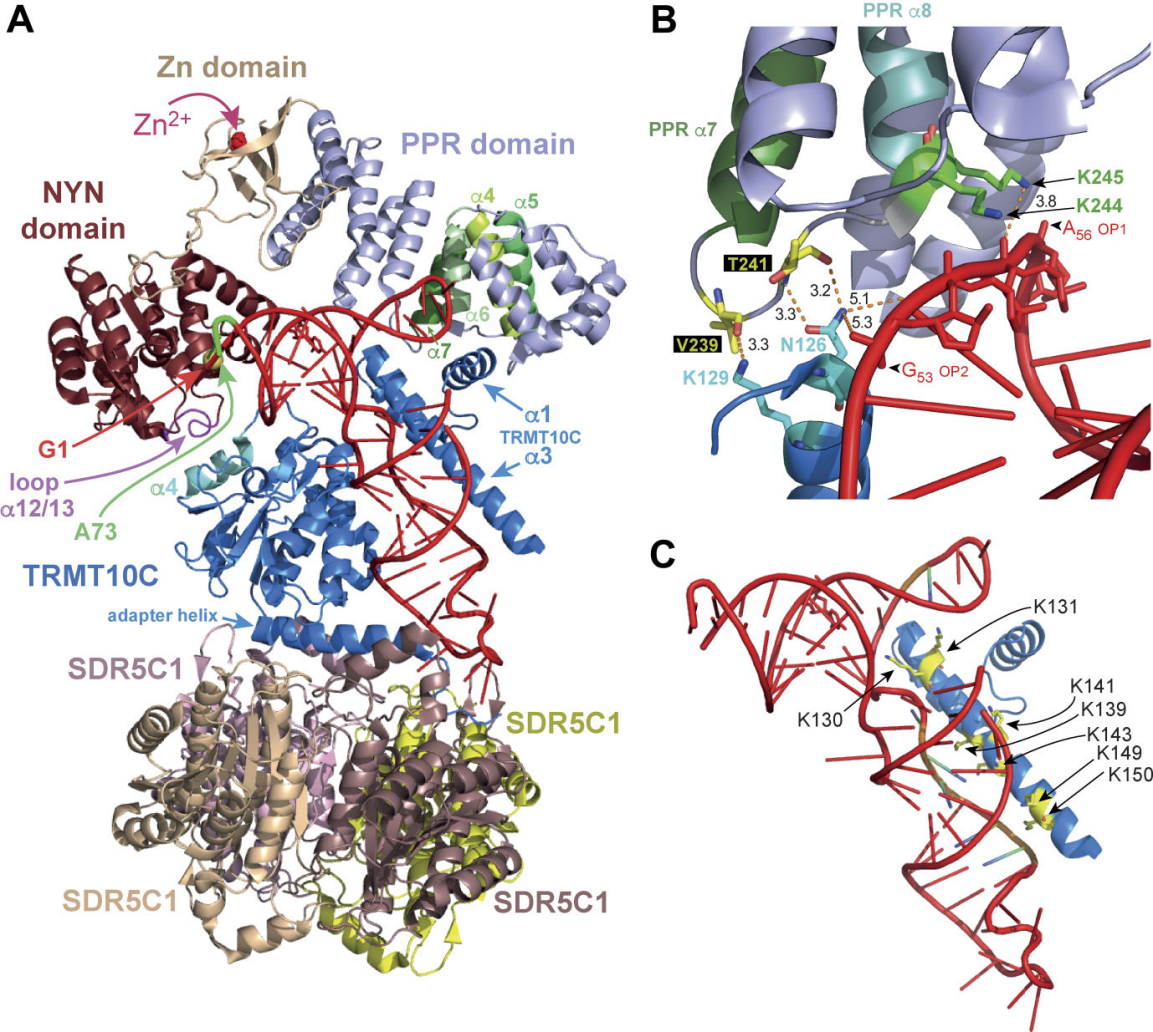
**A**) Cryo-EM structure of human mtRNase P in complex with a human mitochondrial pre-tRNA^Tyr^ (PDB: 7ONU) [22]. The subunits of the holoenzyme and the domains of PRORP are individually colored (adapted from [10]). The following elements are marked: nt G_1_ (red) and A_73_ (green), the Zn^2+^ ion (pink), the loop (in purple) that connects α-helices 12 and 13 of PRORP’s nuclease domain and interacts with α4 (cyan) of TRMT10C’s methyltransferase domain, and α1/α3 of TRMT10C’s N-terminal domain involved in contacts with α4–α7 (highlighted in green tones) of PRORPs PPR domain; also note that the structure revealed density of a Mg^2+^ ion (yellow sphere in the background) close to G_1_ of the tRNA. (**B**) Illustration of N126A and K129A of the N-proximal end of TRMT10C’s helix α3 at the interface to the loop connecting PPR helices α7 and α8 and in vicinity of the T loop; several hydrogen bonding distances (in Å) are indicated. Also marked are PPR domain residues K244/245 close to the phosphate backbone of T loop nucleotides U_54_, U_55_, and A_56_. (**C**) Positively charged side chains in helix α3 of TRMT10C’s N-terminal domain that are poised to interact with the backbone of the tRNA (in red); all seven lysines marked in yellow were substituted by alanines in the TRMT10C variant Kα3A.

In the functional human mtRNase P-substrate complex, PRORP interacts with TRMT10C and the pre-tRNA at two interfaces each [22]. On one side, helices α4–α7 of PRORP’s PPR domain contact helix α1 and the N-proximal end of helix α3 of TRMT10C’s N-terminal domain (NTD), while on the other side the loop connecting helices α12 and α13 of the nuclease domain of PRORP contacts α4 of TRMT10C’s methyltransferase domain (for overview see Fig. 6A). The nuclease domain of PRORP interacts with the acceptor stem and likely the proximal 5′-leader nucleotides, while the PPR domain contacts the T arm. To investigate the influence of the contact region between TRMT10C and the PPR domain on the processing by PRORP, we generated TRMT10C variants intended to weaken or abolish the interaction with the region α4–α7 of PRORP. This included combined substitutions of N126A and K129A at the N-proximal end of TRMT10C’s helix α3 that were inferred to be in contact with the loop connecting PPR helices α7 and α8 (Fig. 6B), and/or deletion of helix α1 (Δα1, see Fig. 6A). The so-called connector helix α3 of TRMT10C clings to a groove in the distorted tRNA structure that extends from the T arm over the D arm and the variable loop to the AC stem (Fig. 6C). The helix is lined with positively charged amino acid side chains that face and contact the tRNA [22]. To assess the importance of this positively charged surface of helix α3 for PRORP catalysis, we simultaneously substituted seven of those tRNA-facing residues with alanines (i.e. lysines 130, 131, 139, 141, 143, 149, and 150; variant Kα3A; Fig. 6C).

**Figure 7.**
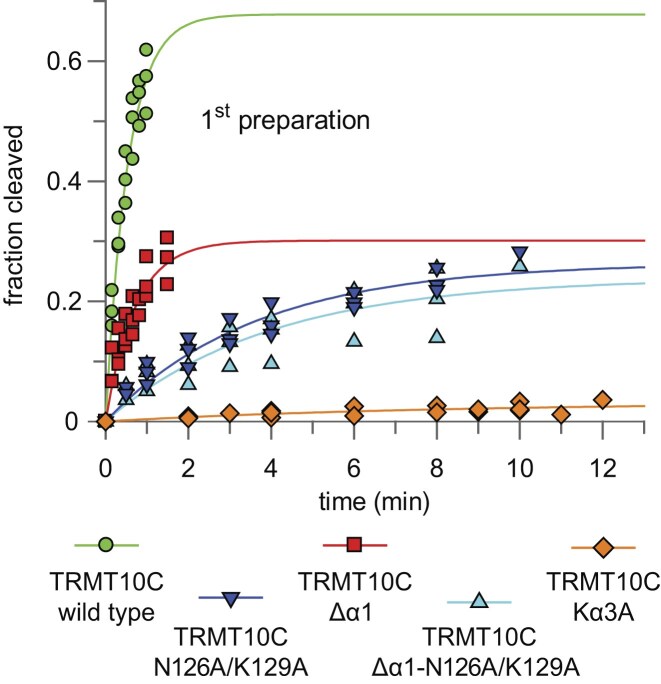
Time courses of *Tt* pre-tRNA^Gly^ (A_-1_/A_44_/A_45_/A_73_; 0.2 nM) processing by human mitochondrial PRORP (100 nM) in the presence of complexes consisting of SDR5C1 and wild-type or variant forms of TRMT10C (200 nM). Recombinant TRMT10C and His-tagged SDR5C1 were combined and complexes affinity-purified using magnetic beads. The data were obtained from three replicate experiments with the first in-parallel preparation of TRMT10C–SDR5C1 complexes (see also Table 6). TRMT10C wild-type; TRMT10C Δα1, TRMT10C lacking helix α1 at its N-terminus; TRMT10C N126A/K129A, TRMT10C with N126A and K129A substitutions; TRMT10C Δα1-N126A/K129A, TRMT10C lacking helix α1 and carrying substitutions N126A and K129A; TRMT10C Kα3A, TRMT10C with seven Lys to Ala substitutions at positions 130, 131, 139, 141, 143, 149, and 150 in helix α3.

We tested the activities of three independent preparations of each TRMT10C variant to take batch-to-batch variability into account, as the reconstitution of recombinant TRMT10C–SDR5C1 complexes was observed to be sensitive to perturbations. We also performed the kinetic analyses at subsaturating PRORP (100 nM) and TRMT10C–SDR5C1 (200 nM) concentrations for better detection of binding defects caused by these TRMT10C alterations, and used the *Tt* pre-tRNA^Gly^ variant A_-1_/A_44_/A_45_/A_73_ for increased cleavage rates. The obtained kinetic data are shown in Fig. 7 and Table 6. The deletion of helix α1 had only a marginal effect on the cleavage rate constant *k*_obs_, but showed substantially reduced endpoints derived from fitting the data to a single exponential (Table 6). This finding is consistent with a role of α1 in stabilizing the TRMT10C–PRORP interaction, without substantially affecting the catalytic activity of PRORP. The lower endpoints thus appear to be attributable to the reduced formation of productive mtRNase P-substrate complexes upon deletion of helix α1. Substitutions N126A/K129A in TRMT10C severely impaired processing by mtRNase P, displaying a 6- to 11-fold decrease in *k*_obs_ compared to wild-type TRMT10C (Table 6). The reaction endpoints were reduced to a similar extent as for the Δα1 variant. Thus, the N126A/K129A negatively affected catalytic efficiency and complex formation. The TRMT10C variant combining the N126A/K129A exchanges and Δα1 behaved kinetically similar to the N126A/K129A variant alone (Table 6), suggesting that residues N126 and K129 of helix α3 play a key role in the conformational activation of PRORP, while the contribution of helix α1 is considerably lower, structurally supportive and essentially silent in the presence of the N126A/K129A double substitution.

**Table 6.** Pre-tRNA processing by PRORP in the presence of TRMT10C variants

TRMT10C variants	First preparation		Second preparation		Third preparation	
	*k*_obs_/*k*_obs*_ (min^−1^)^a^	EP (%)	*k*_obs_/*k*_obs*_ (min^−1^)^a^	EP (%)	*k*_obs_/*k*_obs*_ (min^−1^)^a^	EP (%)
**Wild-type**	1.86 ± 0.25	68 ± 5	1.76 ± 0.13	77 ± 3	2.24 ± 0.24	64 ± 3
**Δα1** ^b^	1.40 ± 0.29	30 ± 3	1.68 ± 0.55	38 ± 6	2.16 ± 0.27	27 ± 1
**N126A/K129A** ^c^	0.28 ± 0.04	26 ± 2	0.16 ± 0.03	49 ± 6	0.36 ± 0.04	39 ± 2
**Δα1-N126A/K129A** ^d^	0.25 ± 0.07	24 ± 3	0.27 ± 0.05	27 ± 2	0.32 ± 0.08	24 ± 3
Kα3A^e^	(2.2 ± 0.6) × 10^−3^		(2.7 ± 0.7) × 10^−3^		(3.2 ± 0.8) × 10^−3^	

Single-turnover rate constants (*k*_obs_) for the processing of *Tt* pre-tRNA^Gly^ (A_-1_/A_44_/A_45_/A_73_) by human mitochondrial PRORP (100 nM) in the presence of wild-type and variant TRMT10C proteins (200 nM) in complex with SDR5C1.

^a^ *k*_obs_ represents mean values ± standard deviation derived from fitting the data to the equation for a single exponential, except for variant Kα3A for which the very low activities could only be quantified by linear fitting (*k*_obs*_). The endpoints (EP) are the limit values (± standard error) obtained for the fits to the single exponential (each mean value based on three replicate experiments). Recombinant TRMT10C and SDR5C1 were combined and preincubated for complex formation, followed by batch-purification of the complex. We tested independent purifications of each complex assembled from SDR5C1 and the respective TRMT10C variant to take batch-to-batch variability into account.

^b^TRMT10C lacking helix α1 at its N-terminus.

^c^TRMT10C with N126A and K129A substitutions.

^d^TRMT10C lacking helix α1 and carrying substitutions N126A and K129A.

^e^TRMT10C with seven Lys to Ala exchanges at positions 130, 131, 139, 141, 143, 149, and 150 in helix α3.

The TRMT10C variant Kα3A showed the most severe activity loss. The seven amino acid substitutions resulted in an ∼650- to 850-fold decrease in cleavage rate under the applied conditions, indicative of PRORP-alone activity (see also Table 4). We conclude that helix α3 is essential for substrate binding by TRMT10C (Table 6), in line with the cryo-EM structure [22].

We also conducted methylation assays to assess the functionality of the modified TRMT10C proteins (Supplementary Fig. S6). The hierarchy of defects was similar to those observed in the mtRNase P cleavage assay (Fig. 7). The methylation activity of the Δα1 variant was almost equal to that of the wild-type TRMT10C protein (Supplementary Table S6), consistent with the major function of helix α1 in structurally stabilizing the TRMT10C–PRORP interaction that is not relevant in the methylation assay performed in the absence of PRORP. Variant Kα3A also exhibited the most severe impairment in methylation activity, with an ∼100-fold decrease in *k*_obs_ compared to wild-type TRMT10C (Supplementary Table S6), further substantiating the crucial role of positively charged amino acids in helix α3 for tRNA binding. The double substitution variant N126A/K129A, with or without additional deletion of helix α1, surprisingly displayed a roughly tenfold reduction in methylation rate.

## Discussion

### Interactions immediately upstream of the cleavage site and longer 5′ extensions stimulate pre-tRNA cleavage

Pre-tRNA-5′ maturation by human mtRNase P appears to be compatible with leaders of a great variety of lengths. When tested with a bacterial model substrate, processing efficiency was similar for lengths of 14 and 7 nt, decreased gradually for 4- and 2-nt long leaders and only sharply dropped for a pre-tRNA carrying a 1-nt leader (Table 1). For human mitochondrial pre-tRNA^Ala^, a similar reduction and drop in cleavage efficiency was observed when the leader was reduced to 1 nt (Supplementary Table S3), yet the effect on *K*_M(sto)_ was markedly different for the two substrates, an ∼2-fold decrease in the case of mitochondrial pre-tRNA^Ala^ contrasting with an ∼9-fold increase for *Tt* pre*-*tRNA^Gly^, relative to their parental pre-tRNAs with 50-nt and 14-nt leaders, respectively. A sharp activity drop was also previously observed in reactions catalyzed by *At*PRORP3 when the 5′-leader was shortened from 2 to 1 nt [32]. With *At*PRORP3 using the same set of *Tt* pre*-*tRNA^Gly^ variants, the 1-nt leader substrate showed an ∼10-fold decrease in *k*_react_ and essentially no *K*_M(sto)_ effect, more similar to mitochondrial pre-tRNA^Ala^ in our current study. In general, the cleavage-rate drop with both enzyme types in the presence of a 1-nt leader can be attributed to the fact that their metallonuclease active sites are adapted to interact with phosphodiesters between nt − 2 and − 1. The latter carry a total of one negative charge distributed on two nonbridging phosphate oxygens, while a terminal 5′-phosphate at this position introduces two negative charges. Specifically, human PRORP was recently found to interact with nt − 1, −2, and even − 3 of the pre-tRNA^His^ leader [23], well in agreement with our results. The differential effect on *K*_M(sto)_ for the cleavage of the two substrates by mtRNase P, however, points to effects conferred by the different tRNA bodies, possibly related to the structural rigidity and, as a result, reduced conformational malleability of the tRNA^Gly^.

Our previous [32] and present (Supplementary Table S4) results obtained with single-polypeptide *At*PRORP3 suggested that the enzyme does not form intimate contacts with leader nucleotides between positions −2 and −55. For human mtRNase P, the data presented here suggest that cleavage efficiency is even higher with leaders longer than 7–14 nt, indicative of leader contacts at some distance to the cleavage site. This effect was observed with three different pre-tRNAs, but is unexpected, considering that such long leaders may interfere with tRNA folding equilibria (Supplementary Fig. S7). A possible explanation for this difference between mtRNase P and *At*PRORP3 could be the different surface charge distribution of human PRORP and *At*PRORPs; human PRORP displays distinguishable and expanded positive patches, particularly in comparison with *At*PRORP2 (Supplementary Fig. S8), the nuclear RNase P isoenzyme of *A. thaliana* beyond *At*PRORP3. These positive patches may form an extended swath for leader binding on the surface of human PRORP (Supplementary Fig. S8A). While such hypothesis is currently speculative, the effect of a longer leader appears also dependent on the linked tRNA body and was, for example, more pronounced in the case of mitochondrial tRNA^Tyr^ than of bacterial tRNA^Gly^. In the case of pre-tRNA^Ala^, an increase of the leader beyond 50 nt, to include the complete upstream tRNA^Asn^, resulted in a moderate reduction in cleavage efficiency, likely because of competition for enzyme binding by the additional tRNA structure.

Although the number of leader variants analyzed here is too low to draw final conclusions, our findings are consistent with the “tRNA punctuation model” of human mitochondrial transcript maturation, according to which mtRNase P initiates the processing of long polycistronic transcripts by cleavage at the 5′-ends of tRNAs interspersed between messenger RNAs (mRNAs) and ribosomal RNAs (rRNAs).It is therefore tempting to speculate that human mtRNase P specifically evolved to act efficiently on mitochondrial tRNA structures embedded in long native primary transcripts. At first sight, mitochondrial tRNA^Ala^ appears to be in an unfavorable natural position in this regard, being separated from the upstream tRNA^Asn^ by only 1 nt. However, the still efficient processing of the tRNA^Asn^-tRNA^Ala^ tandem (74-nt leader variant in Supplementary Fig. S1A and Supplementary Table S3) suggests that mtRNase P cleavage of pre-tRNA^Ala^ may naturally well precede 3′-end cleavage of pre-tRNA^Asn^ by RNase Z and thereby largely avoid the unfavorable 1-nt-leader situation.

### Nucleotide identities at the cleavage site and the accessibility of the scissile bond modulate catalysis

In many bacteria and archaea, a U residue is predominantly found at position −1 of pre-tRNA leaders [47]. The conserved residue A248 in the catalytic center of bacterial RNase P RNA forms a parallel stacking interaction with the U_−1_ and the G_1_-C_73_ base pair of the pre-tRNA [48]. The highest cleavage rate by mtRNase P was observed in the presence of an A at −1. The maximum cleavage rate (*k*_react_) was 2.4-fold higher for pre-tRNA^Gly^ with A_−1_ versus C_−1_ (Table 3), an increase similar to that previously observed with *At*PRORP3 (∼3-fold) [32]; a consistent, but less pronounced increase in cleavage rate (*k*_obs_) was also observed when the U_−1_ of human mitochondrial pre-tRNA^Ala^ was changed to A_−1_ (Supplementary Table S5). However, unlike nucleus-encoded tRNAs of *A. thaliana*, where A is most commonly found at position −1, U_−1_ is more common upstream of tRNA genes in the human mitochondrial genome (10 of 22) than A_−1_ (7 of 22). Notably, in 5 of the 10 cases this U_−1_ encodes the first position of an incomplete UAA stop codon of an upstream mRNA to be completed by polyadenylation. These observations suggest that adapting the base identity at position −1 to optimize mtRNase P cleavage efficiency has not been a dominant constraint in human mitochondrial genome evolution, but other evolutionary pressures have been (counter)acting on the identity of this position.

*Tt* pre-tRNA^Gly^ and mitochondrial pre-tRNA^Ala^ variants with an extra G_−1_-C_73_ base pair showed small reductions in cleavage efficiency (Table 3 and Supplementary Table S5). This kinetic disadvantage is likely due to the restricted accessibility of the canonical phosphodiester, consistent with aberrant cleavage at the −2/−1 phosphodiester (see below) and the absence of G_−1_-C_73_ or C_−1_-G_73_ base pairs in human mitochondrial tRNAs. The reason for the slightly reduced cleavage efficiency of a U_1_-A_72_ variant of *Tt* pre-tRNA^Gly^ is less clear and human mitochondrial tRNAs for Arg, Asn and Gln have such a terminal acceptor-stem base pair. Noteworthy, the same pre-tRNA^Gly^ variant carrying a U_1_-A_72_ base pair at the cleavage site showed essentially unchanged catalytic efficiency relative to the parental substrate in reactions catalyzed by *At*PRORP3 [32]. Overall, we conclude that the microenvironments of the metallonuclease catalytic center are similar in single-polypeptide PRORPs and metazoan mtRNase P complexes, although the two enzyme subtypes react in part differentially to substrate alterations in the immediate vicinity of the cleavage site. One explanation could be that the energetic contribution of individual RNA–protein contacts to substrate positioning and transition state stabilization vary between the two enzyme subtypes.

### The interaction with TRMT10C–SDR5C1 restricts the measuring flexibility of PRORP

Intriguingly, TRMT10C–SDR5C1 appears to strongly constrain the flexibility of human PRORP in cleavage-site positioning. Whereas the cleavage of the *Tt* pre-tRNA^Gly^ variant with an extra G_−1_-C_73_ base pair is entirely redirected to 1 nt upstream (between positions −1 and −2) in reactions catalyzed by *At*PRORP3 or by human PRORP (without TRMT10C–SDR5C1), the mtRNase P holoenzyme cleaved this substrate still mostly at the canonical site, with only 25% of miscleavage (Fig. 2); with the corresponding G_−1_-C_73_ variant of mitochondrial pre-tRNA^Ala^ this effect was in principle similar, though less pronounced (Supplementary Fig. S5). On the one hand, this underlines the evolutionary relationship of human PRORP to its single-subunit relatives. On the other hand, the finding confirms the role of TRMT10C–SDR5C1 in the positioning of the target phosphodiester bond in the active site of PRORP in addition to or as part of its rate-enhancing functions [15]. The more rigid measuring mechanism of the mtRNase holoenzyme in cleavage-site selection might result from its firmer hold of tRNAs, which evolved to cope with the structurally heterogeneous and conformationally labile ensemble of mitochondrial tRNAs. Although this entails the capacity to at least partially expose the canonical cleavage site in the presence of a G_−1_-C_73_ base pair, such base pairs are nevertheless avoided in human mitochondrial pre-tRNAs, possibly because processing exclusively at the canonical site is crucial for tRNA 5′-end and mRNA 3′-end formation alike in mitochondrial transcript maturation.

Nucleotide identity at, and base pairing upstream of the cleavage site, as well as inhibition through nearby RNA structures play a role in the kinetic hierarchy of the RNase P processing pathways for polycistronic/multimeric tRNA transcripts in bacteria [48, 56, 57]. In principle, similar mechanisms may also play a role in “ordering” processing pathways of clustered mitochondrial tRNAs. However, in contrast to bacteria, with their homogenously canonical tRNAs, the heterogeneity of mitochondrial tRNA structures themselves has to be assumed to also play a crucial, possibly dominant role. Notably, mitochondrial pre-tRNAs have been observed to be processed at unusually differing rates [15], which appears only in part explainable by leader and −1-nt identity. The pre-tRNA^His^-tRNA^Ser(AGY)^-tRNA^Leu(CUN)^ cluster appears to exemplify this: (i) lack of the D arm apparently prevents TRMT10C–SDR5C1 binding and thereby appreciable cleavage by PRORP (Fig. 3); (ii) in crude mitochondrial extracts 5′-end cleavage of pre-tRNA^His^ appears kinetically favored over that of pre-tRNA^Leu(CUN)^ despite a suboptimal U_−1_ in the former and an optimal A_−1_ in the latter (see Fig. 2 in [34]).

### MtRNase P interacts with all parts of the tRNA structure but the interactions with the anticodon loop are of minor significance

RNA-based forms of RNase P from all three domains of life as well as single-subunit PRORPs interact with the coaxially stacked acceptor stem/T domain but not with any other subdomain of the tRNA body [1, 6, 10]; consistently, these enzyme forms are generally able to efficiently cleave the 5′ leader from pre-tRNA substrates lacking the D and AC domain, and natural mimics thereof. Human mtRNase P, in contrast, interacts with all four domains of the tRNA body [22]. The extra interactions are due to the acquisition of TRMT10C–SDR5C1 as an accessory subunit, and human PRORP itself contacts essentially the same tRNA elements as its single-subunit relatives. Consistently, the mtRNase P holoenzyme responds with a dramatic drop in cleavage rate to the deletion of either the D or the D and the AC arm, whereas PRORP alone responds with a much lower drop in cleavage rate to the deletion of the two domains, albeit starting from a lesser cleavage capability for the intact pre-tRNA molecule. This demonstrates that the strongly rate-enhancing function of the TRMT10C–SDR5C1 complex critically depends on its interaction with the tRNA body, and even high concentrations of the complex cannot support the nuclease activity of PRORP in the absence of simultaneous TRMT10C–SDR5C1 contacts to the same pre-tRNA substrate. This extra tRNA-binding subcomplex has likely been an important evolutionary acquisition for maintaining tRNA 5′-end maturation despite the degeneration of canonical structural features in metazoan mitochondrial tRNAs [10, 15]. However, the compensation of the accompanying erosion of the specificity in the interactions of PRORP with the pre-tRNA through its interactions with TRMT10C apparently reached its limits (see Table 4) in the case of the D armless mitochondrial tRNA^Ser(AGY)^. Efficient processing of this tRNA could apparently only be secured by hooking it to the 3′ end of another tRNA gene to indirectly achieve 5′-end processing through RNase Z cleavage at the 3′ end of the adjoining tRNA as already proposed 25 years ago [34].

Unlike the D and T loops, the AC loop of metazoan mitochondrial tRNAs has maintained the conserved size of 7 nt with base identities at specific positions comparable to those of canonical tRNAs. It seems therefore plausible that human mtRNase P utilizes the AC domain to recognize the diverse set of human mitochondrial tRNAs. The cryo-EM structure of human mtRNase P revealed the involvement of four stacking interactions in the binding of the AC loop of mitochondrial tRNA^Tyr^: A_36_:A_38_, U_33_:A_37_ as well as TRMT10C R185:C_32_ and F177:U_35_ (Fig. 4A) [22]. In addition, the terminal NH_2_ group of TRMT10C residue R181 can form an H bond (2.4 Å) with the *O*^2^oxygen of U_33_; the guanidinium group of R181 is further positioned for bifurcated H bonding with the OP1 oxygens of G_34_ and A_37_ of tRNA^Tyr^ (3.6 and 3.4 Å) to bridge the loop backbone (Fig. 4B). The 2′-OH group of A_37_ is also within reach of the *O*^2^of U_33_ (3.3 Å) and close to the terminal NH_2_ group of R181 (4.1 Å). This potential H-bonding network involving R181, together with the four aforementioned stacking interactions, is suggested to stabilize the specific AC loop conformation in the bound state. When we exchanged the pyrimidine at position 33 with an A residue, we indeed observed an ∼2.5-fold reduction of *k*_max_ and an ∼2-fold decrease in *k*_max_/*K* (Table 5). The effect is consistent with the disruption of H bonding between R181 and the *O*^2^of U_33_ and may also include a steric effect due to introducing a bulkier purine at this position where human mitochondrial tRNAs exclusively carry a pyrimidine (mostly U; C in tRNA^Met^, Ψ in tRNA^Gln^). The kinetic effect of U_33_→A_33_, which is remarkable considering that the AC loop is located furthest from the cleavage site, would be in line with the idea that the contact between Y_33_ (preferentially U) and R181 of TRMT10C represents a main anchor point for docking the AC stem-loop to the enzyme. Other AC alterations (U_32_→A_32_, G_34_→U_34_ and A_38_→C_38_; Table 5) had no or minor effects. The ∼2-fold reduced *K*-values and little effect on *k*_max_ for variants A_32_ and C_38_ can be assigned to the elimination of the U_32_:A_38_ base pair at the beginning of the AC loop. Such base pairing likely increases the activation energy barrier for the structural transition of the AC loop during binding to TRMT10C–SDR5C1, which requires A_38_ to swivel out for stacking with A_36_. As part of the AC triplet, position 34 is not conserved in tRNAs, consistent with our observation that a G_34_→U_34_ exchange was kinetically neutral. Based on the cryo-EM structure, several potential interactions of G_34_ of mitochondrial tRNA^Tyr^ with one SDR5C1 protomer were proposed, including an H bond of the ε-NH_2_ group of K105 to the *N*^7^ of G_34_ (3.7 Å) [22]. Yet, this ε-NH_2_ group has the same distance to the OP2 of U_33_, leaving the possibility that H bonding to the latter may prevail. S98 and Q107 with H-bonding donor capacity of their terminal side chain groups are close to G_34_ as well, but their contribution to interaction with bases at position 34 remains unclear.

The kinetic curves for TRMT10C–SDR5C1 titration at near-saturating PRORP concentration (500 nM) showed sigmoidal behavior (Fig. 5). This behavior was observed consistently across all the AC loop variants, albeit with different extents of *S*-shape curvature. The inferred cooperativity might reflect the concentration-dependent binding equilibria involved in complex assembly, and particularly be related to the 2:4 stoichiometry of the TRMT10C–SDR5C1 complex [20], resulting in a “bivalent,” mirror symmetric structure able to bind 2 pre-tRNA molecules [10, 23]; still, other causes of sigmoidicity cannot be entirely excluded at present.

### The interactions of TRMT10C with the tRNA are crucial for catalysis by PRORP

Regarding the tested TRMT10C variants, we have provided evidence that helix α1 (i) stabilizes the TRMT10C–PRORP interaction without affecting the catalytic activity of mtRNase P and (ii) that the absence of helix α1 has only a minor effect on TRMT10C’s methylation activity (Fig. 7 and Table 6, and Supplementary Fig. S6 and Supplementary Table S6). The cleavage and methylation activities in the presence of the TRMT10C variant Kα3A showed decreases in the order to 2 to 3 orders of magnitude, in line with the key function of positively charged amino acid side chains in helix α3 for tRNA recognition, in line with the cryo-EM structure [22].

The cryo-EM structure also suggests that N126 and K129 at the N-proximal end of helix α3 form contacts with V239 and T241 in the loop connecting PPR helices α7 and α8 of PRORP (Fig. 6B). Combined N126A and K129A substitutions severely impaired the cleavage activity of mtRNase P, with little additional effect upon further deletion of helix α1 (Fig. 6A and Table 6). This finding is consistent with the function of the K129:V239 and N126:T241 contacts in adjusting the orientation of PRORP’s PPR domain as suggested by the cryo-EM structure. However, the strongly impaired methylation activity of TRMT10C N126A/K129A (with or without helix α1) was surprising, considering that PRORP was absent from methylation assays and is dispensable for methylation [14]. A possible explanation could be that N126 and K129 are directly involved in contacts to residues of tRNA T arms, either in initial encounter complexes, or persistently during R9 methylation reactions in the absence of PRORP. Conceivably, such direct tRNA contacts involving N126/K129 might be converted to contacts with PRORP’s loop connecting PPR helices α7 and α8 during mtRNase P assembly. The distance of the terminal amino group of N126 is only 5.1 Å to the OP2 of U_54_ and 5.3 Å to the OP1 of G_53_ (Fig. 6B) [22], consistent with such a scenario. Also, human TRMT10A and B, as well as *A. thaliana* and *Saccharomyces cerevisiae* Trm10 carry an N, R, or K residue at the position corresponding to N126 of TRMT10C; likewise, residues corresponding to K129 are K or R in the aforementioned methyltransferases (22, Supplementary Note S2 therein). This broad conservation of the two side chains with H bonding donor functions also in related methyltransferases that do not interact with PRORPs would be consistent with their role in contacts to the tRNA.

Overall, our findings substantiate the encasement of the entire tRNA moiety by human mtRNase P, which sets the enzyme apart from all other known forms of RNase P enzymes including the related single-polypeptide PRORPs [5, 6, 10, 58]. Moreover, we provide the first details on the contribution of individual structural RNA and protein elements to the productive processing of pre-tRNA substrates by mtRNase P.

## Supplementary Material

gkaf1145_Supplemental_Files

## Data Availability

The data underlying this article are available in the article and in its online supplementary material. Primary data underlying enzyme kinetic analyses will be shared on reasonable request to the corresponding author.
