# Supplementary material for: Substrate and enzyme determinants for recognition by human mitochondrial RNase P
Source: Nucleic Acids Res. 2025 Nov 20;53(21):gkaf1145. doi: 10.1093/nar/gkaf1145 (PMC12630138; doi:10.1093/nar/gkaf1145)
Supplement: gkaf1145_Supplemental_Files [file gkaf1145_supplemental_files.zip › Hazisllari et al 2025 - rev Supplementary Information.pdf]

SUPPLEMENTARY DATA

Substrate and enzyme determinants for recognition by human mitochondrial RNase P

Enxhi Hazisllari[1], Danijela Radovanović[1], Ursula Toth[1], Elisa Vilardo[1], Roland K. Hartmann[2] and Walter Rossmanith[1,*]

[1]Center for Anatomy & Cell Biology, Medical University of Vienna, 1090 Vienna, Austria

[2]Institute of Pharmaceutical Chemistry, Philipps-University Marburg, 35037 Marburg, Germany

[*]To whom correspondence should be addressed.
Tel: +43 1 40160 37512; Email: walter.rossmanith@meduniwien.ac.at

Supplementary Table S1. Primers used for site-directed mutagenesis to generate TRMT10C variants. This table can be found in the accompanying Excel file.

Supplementary Table S2. PCR primers used for to generate the templates for *in vitro* transcription of pre-tRNAs. This table can be found in the accompanying Excel file.

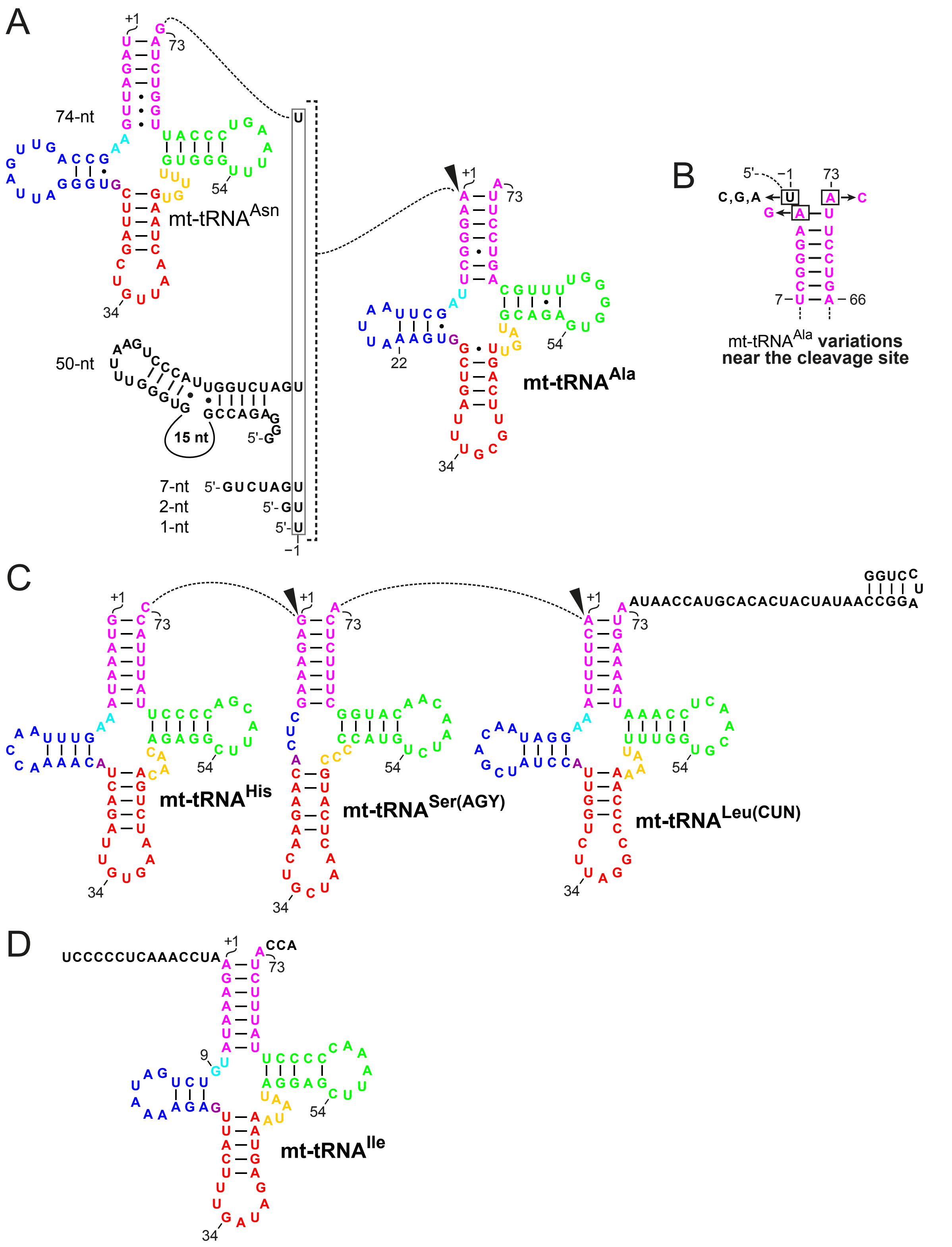

Supplementary Figure S1. Sequences and (predicted) secondary structures of additional substrates analyzed in this study. (**A**) 5'-leader variants of human mitochondrial pre-tRNA^{Ala}, with different leader sequences indicated on the left. The structural elements of the tRNA body are color-coded: magenta, aminoacyl acceptor stem; blue, D arm; red, anticodon arm; gold, variable loop; green, T arm. The positions of selected nucleotides are numbered according to convention (1). The canonical RNase P cleavage site (black arrowhead) is located between nucleotides −1 and +1. (**B**) Nucleotide exchanges near the RNase P cleavage site analyzed in the context of mitochondrial pre-tRNA^{Ala}. (**C**) Precursor

encoding human mitochondrial $tRNA^{His}$, $tRNA^{Ser(AGY)}$ and $tRNA^{Leu(CUN)}$, starting with the 5'-terminal nt of $tRNA^{His}$ and terminating 33 nt downstream of $tRNA^{Leu(CUN)}$. (**D**) The human mitochondrial pre-$tRNA^{Ile}$ used for methylation experiments in this study.

Supplementary Table S3. Role of 5'-leader length variation in mitochondrial pre-tRNA^{Ala} processing by human mtRNase P

	k_{obs} (min^{-1})[a]	k_{react} (min^{-1})[b]	$K_{M(sto)}$ (nM)[b]
**50-nt leader**	0.50 ± 0.02	0.51 ± 0.05	91 ± 23
**7-nt leader**	0.20 ± 0.07		
**2-nt leader**	0.13 ± 0.03		
**1-nt leader**	0.06 ± 0.01	0.10 ± 0.01	42 ± 18
**74-nt leader**[c]	0.28 ± 0.03	0.29 ± 0.02	79 ± 20

Single-turnover rate constants for the processing of human mitochondrial pre-tRNA^{Ala} with different 5'-leader lengths and with the discriminator nucleotide A_{73} as the 3'-end. Substrates variants were cleaved by PRORP in the presence of TRMT10C-SDR5C1.

[a]k_{obs} was determined at 500 nM PRORP and 200 nM TRMT10C-SDR5C1 (mean ± standard deviation derived from fitting the data to the equation for a single exponential, based on 21 replicates for the 50-nt leader variant and 5 to 9 replicate experiments each for the other substrates).

[b]The maximal rate constant k_{react} and the enzyme concentration at which the half-maximal rate constant is achieved $K_{M(sto)}$ were determined by a Michaelis-Menten-like kinetics model, utilizing trace amounts of substrate and increasing concentrations of PRORP at a constant concentration of 200 nM TRMT10C-SDR5C1 (best-fit values ± standard error based on 4 to 9 replicate experiments for each PRORP concentration).

[c]Encoding the complete mitochondrial tRNA^{Asn} and a 1-nt spacer.

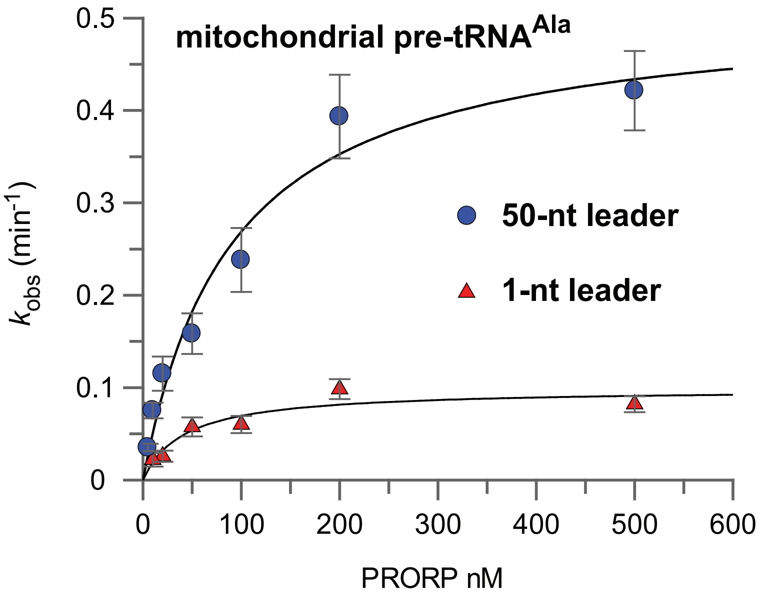

Supplementary Figure S2. Single-turnover kinetics of the processing of mitochondrial pre-tRNA^{Ala} with a 50-nt leader compared to a 1-nt leader by human mtRNase P as a function of PRORP concentration in the presence of 200 nM TRMT10C-SDR5C1; see legend to Supplementary Table S3 for further details and the derived kinetic constants.

Supplementary Table S4. The role of 5'-leader and 3'-trailer length variations on processing of *Tt* pre-$tRNA^{Gly}$ variants by *At*-PRORP3

	6-nt trailer[a]	no trailer[b]
	k_{obs} (min^{-1})	k_{obs} (min^{-1})
14-nt leader	3.00 ± 0.25	4.66 ± 0.02
50-nt leader of pre-$tRNA^{Ala}$	5.92 ± 0.42	4.57 ± 0.25
55-nt leader of pre-$tRNA^{Tyr}$	2.47 ± 0.13	2.79 ± 0.31

Single-turnover rate constants (k_{obs}) for the processing of *Tt* pre-$tRNA^{Gly}$ variants by *At*PRORP3 as a function of 5'-leader length and presence or absence of a trailer sequence, determined at 5 nM PRORP3 (mean ± standard deviation derived from fitting the data to the equation for a single exponential, based on 5 to 6 replicate experiments each).

[a]*Tt* pre-$tRNA^{Gly}$ carrying a trailer of 6 nucleotides (U_{73}-CCAGUC-3' including the CCA sequence).

[b]The aminoacyl acceptor stem carries only of the discriminator nucleotide (U_{73}) at its 3' end.

Supplementary Table S5. The effect of varying base identity at the cleavage site of pre-$tRNA^{Ala}$

	k_{obs} (min^{-1})[a]	k_{react} (min^{-1})[b]	$K_{M(sto)}$ (nM)[b]
pre-$tRNA^{Ala}$ (U_{-1}, A_{73}, A_1, U_{72})	0.50 ± 0.02	0.51 ± 0.05	91 ± 23
C_{-1}	0.48 ± 0.05		
G_{-1}	0.62 ± 0.04		
A_{-1}	0.75 ± 0.06		
G_1	0.62 ± 0.04		
G_{-1}, C_{73}	0.24 ± 0.01	0.31 ± 0.02	68 ± 12

Single-turnover kinetic constants for processing of human mitochondrial pre-$tRNA^{Ala}$ variants (50-nt leader with only discriminator nucleotide A_{73} at the 3'-end) with different nucleotide identities at positions −1, +73, +1 and/or +72 by human mitochondrial PRORP in the presence of TRMT10C-SDR5C1.

[a]k_{obs} was determined at 500 nM PRORP and 200 nM TRMT10C-SDR5C1 (mean ± standard deviation derived from fitting the data to the equation for a single exponential; based on 9 to 22 replicate experiments each).

[b]k_{react} and $K_{M(sto)}$, see legend to Table Supplementary S3 (based on 5 replicate experiments for each PRORP concentration).

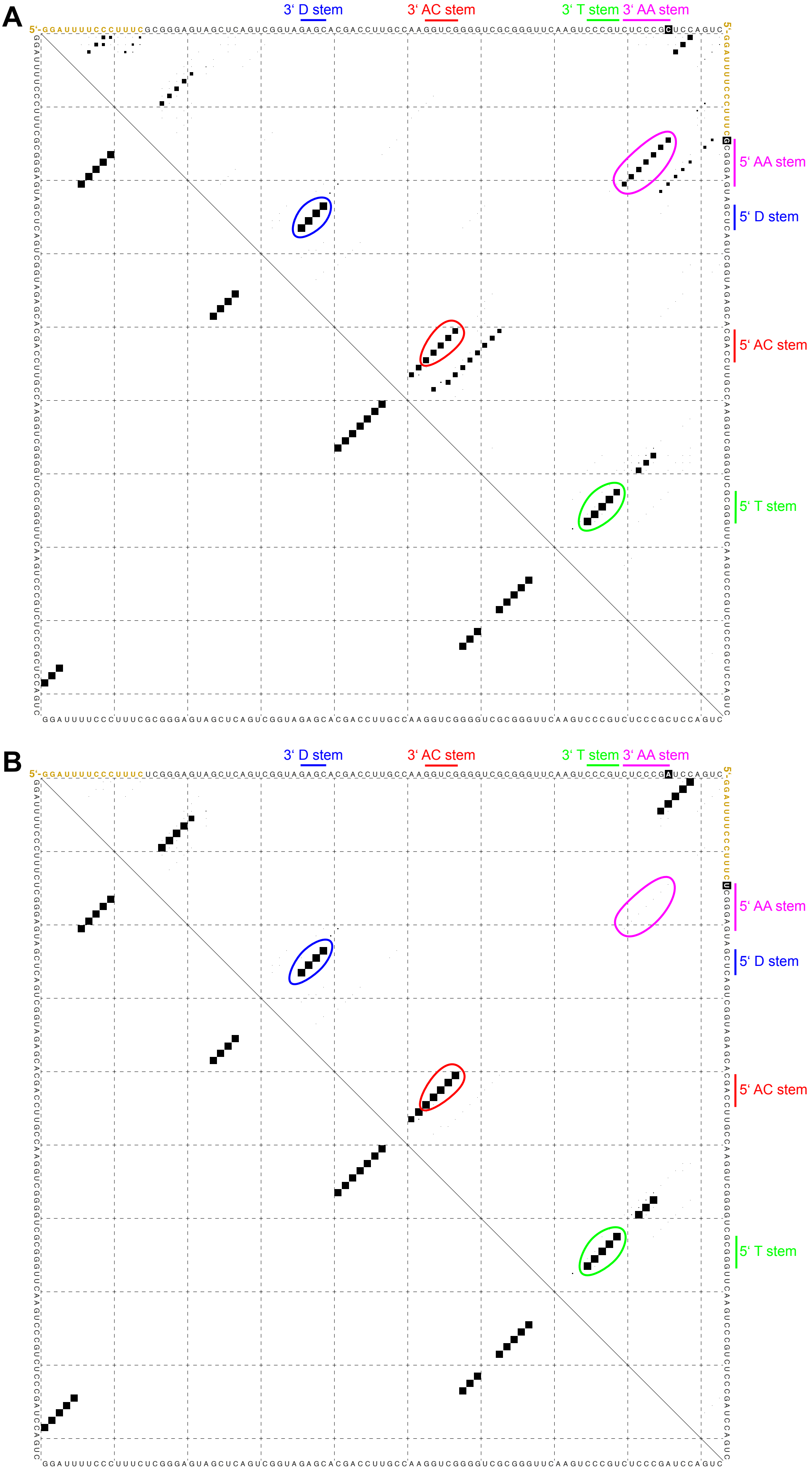
A
3' D stem
3' AC stem
3' T stem
3' AA stem
5' AA stem
5' D stem
5' AC stem
5' T stem
B
3' D stem
3' AC stem
3' T stem
3' AA stem
5' AA stem
5' D stem
5' AC stem
5' T stem

Supplementary Figure S3. Dot plots of RNA secondary structure predictions by *RNAfold* (2) for (**A**) wild-type *Tt* pre-tRNA^{Gly} with its 14-nt leader and 6-nt trailer (U_{73}-CCAGUC-3' including the CCA sequence) (see also Figure 1) and (**B**) the same pre-tRNA but with an U_1-A_{72} base pair instead of G_1-C_{72}. The dots representing the base pairs of the four tRNA stems (AA, aminoacyl acceptor; AC, anticodon) are encircled and colored as in Figure 1 and Supplementary Figure S1; the 5'-leader nucleotides are colored brown and the positions involved in the first base pair are highlighted by black rectangles. The dot pattern below the diagonal represents the minimum free energy (MFE) structure, whereas that above the diagonal illustrates the diversity of the ensemble of predicted near-isoenergetic structures.

The dot plot analysis indicates that replacement of the G_1-C_{72} base pair with U_1-A_{72} reduces the representation of conformers with a regular acceptor stem. Instead, alternative pairings between leader residues, CCA end and nucleotides of both acceptor strands are predicted to be present in the energetically most favorable structures. This prediction would be consistent with the lower experimental endpoint (~85% ± 3%) obtained for the *Tt* pre-tRNA^{Gly} variant with an U_1-A_{72} base pair, explainable by the presence of substrate conformers that lack the acceptor stem, which prevents the formation of productive enzyme-substrate complexes.

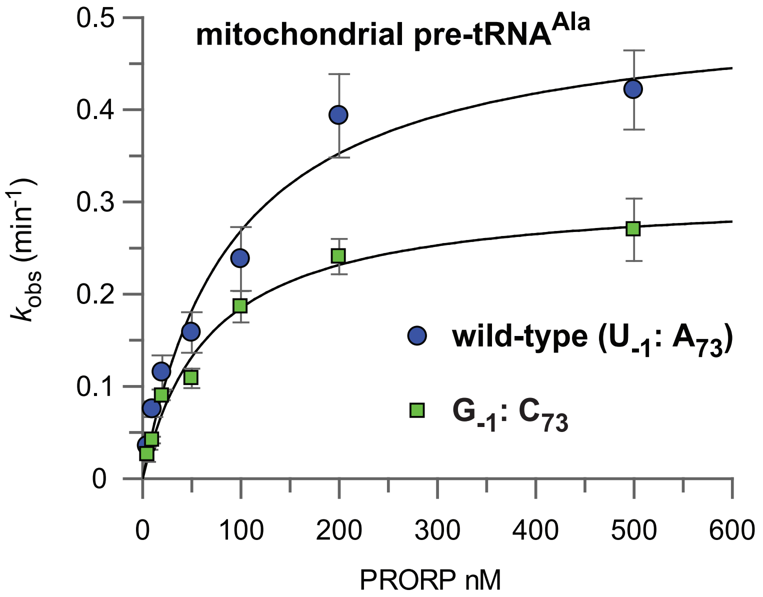

Supplementary Figure S4. Single-turnover kinetics of the processing of mitochondrial pre-tRNA^{Ala} with its natural U_{-1}-A_{73} base pair compared to pre-tRNA^{Ala} with a G_{-1}-C_{73} base pair by human mtRNase P as a function of PRORP concentration in the presence of 200 nM TRMT10C-SDR5C1; see legend to Supplementary Table S5 for further details and the derived kinetic constants.

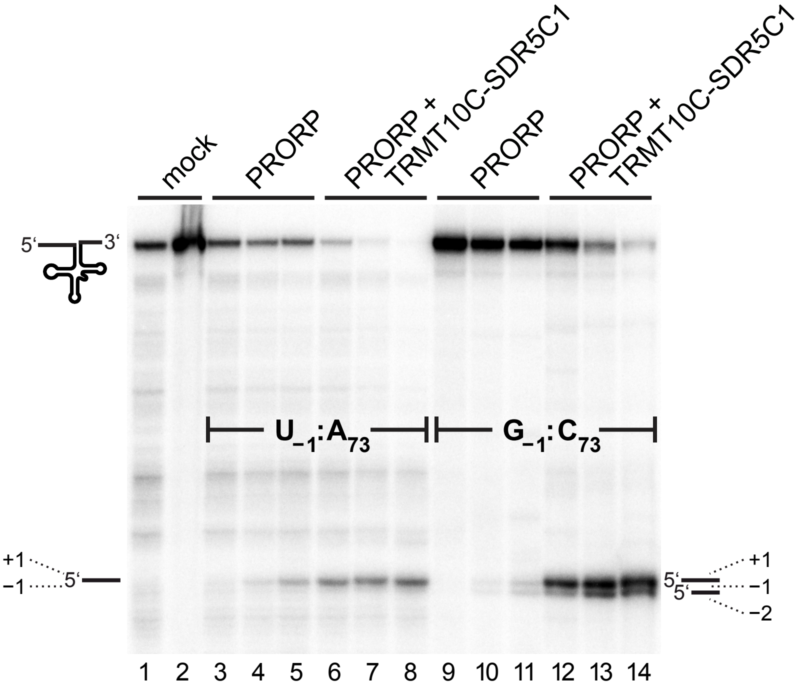

Supplementary Figure S5. Analysis of cleavage site selection by human PRORP alone (500 nM), the mtRNase P holoenzyme (500 nM PRORP + 600 nM TRMT10C-SDR5C1) when acting on mitochondrial pre-tRNA^{Ala} with its natural U_{-1}:A_{73} base pair (lanes 3 to 8) compared to pre-tRNA^{Ala} with a G_{-1}:C_{73} base pair (lanes 9 to 14). Aliquots were withdrawn from the reactions after 1 min (lanes 3, 6, 9, and 12), 10 min (lanes 4, 7, 10, and 13) and 100 min (lanes 5, 8, 11, and 14), respectively. Reaction products were resolved by 6% denaturing PAGE (24:1 acrylamide:bis-acrylamide).

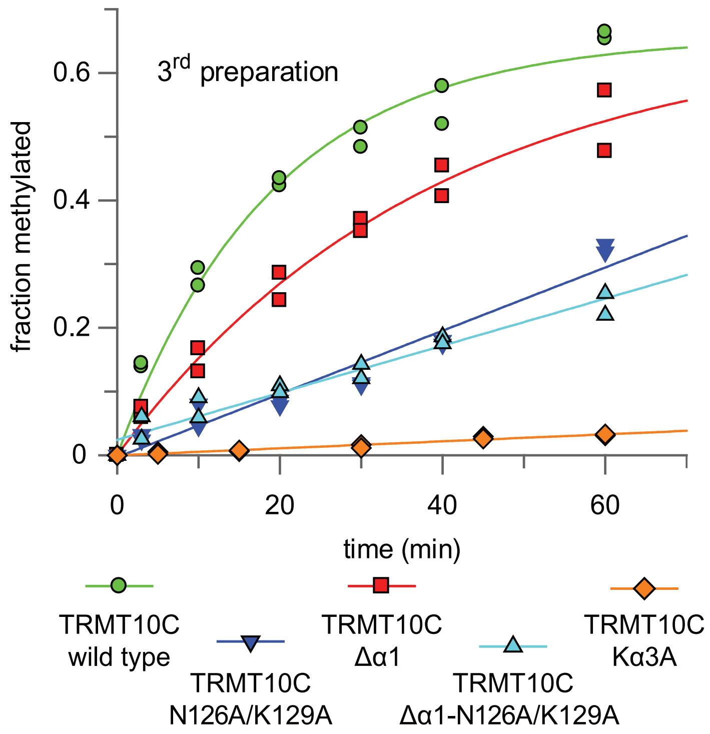

Supplementary Figure S6. Time courses of human mitochondrial pre-tRNA^{Ile} (1 nM) methylation at position 9 by complexes (200 nM) consisting of SDR5C1 and wild-type or variant forms of TRMT10C. Recombinant TRMT10C and His-tagged SDR5C1 were combined and complexes affinity-purified using magnetic beads. The data were obtained from 2 replicate experiments with the 3rd preparation (see also Supplementary Table S6). TRMT10C wild type; TRMT10C Δα1, TRMT10C lacking helix α1 at its N-terminus; TRMT10C N126A/K129A, TRMT10C with N126A and K129A substitutions; TRMT10C Δα1-N126A/K129A, TRMT10C lacking helix α1 and carrying substitutions N126A and K129A; TRMT10C Kα3A, TRMT10C with seven Lys to Ala substitutions at positions 130, 131, 139, 141, 143, 149 and 150 in helix α3.

Supplementary Table S6. Methylation activity of TRMT10C variants

TRMT10C variants	1st preparation		2nd preparation		3rd preparation	
	k_{obs}/k_{obs*} (min^{-1})[a]	EP (%)	k_{obs}/k_{obs*} (min^{-1})[a]	EP (%)	k_{obs}/k_{obs*} (min^{-1})[a]	EP (%)
wild type	0.034 ± 0.005	67 ± 5	0.033 ± 0.003	64 ± 3	0.053 ± 0.006	66 ± 3
Δα1[b]	0.043 ± 0.002	55 ± 1	0.024 ± 0.007	68 ± 11	0.026 ± 0.004	66 ± 6
N126A/K129A[c]	$(3.6 \pm 0.4) \times 10^{-3}$		$(3.8 \pm 0.2) \times 10^{-3}$		$(5.0 \pm 0.3) \times 10^{-3}$	
Δα1-N126A/K129A[d]	$(3.3 \pm 0.2) \times 10^{-3}$		$(4.2 \pm 0.3) \times 10^{-3}$		$(3.7 \pm 0.2) \times 10^{-3}$	
Kα3A[e]	$(5 \pm 2) \times 10^{-4}$		$(3.0 \pm 0.1) \times 10^{-4}$		$(6.0 \pm 0.3) \times 10^{-4}$	

Single-turnover rate constants for the m^1G9 methylation of human mitochondrial pre-tRNA^{Ile} (Supplementary Figure S1) by wild-type TRMT10C and its variants in complex with SDR5C1.
[a]k_{obs} represents mean values ± standard deviation derived from fitting the data to the equation for a single exponential in the case of wild-type and Δα1 TRMT10C, or by linear fitting in the case of TRMT10C variants N126A/K129A, Δα1-N126A/K129A and Kα3A (k_{obs*}). The endpoints (EP) are the limit values (± standard error) obtained for the fits to the single exponential (each mean value based on 2 replicate experiments). The concentration of SDR5C1 complexes with wild-type and variant forms of TRMT10C was adjusted to 200 nM in all experiments. For further details, see also legends to Table 6 and Figure 7.

A

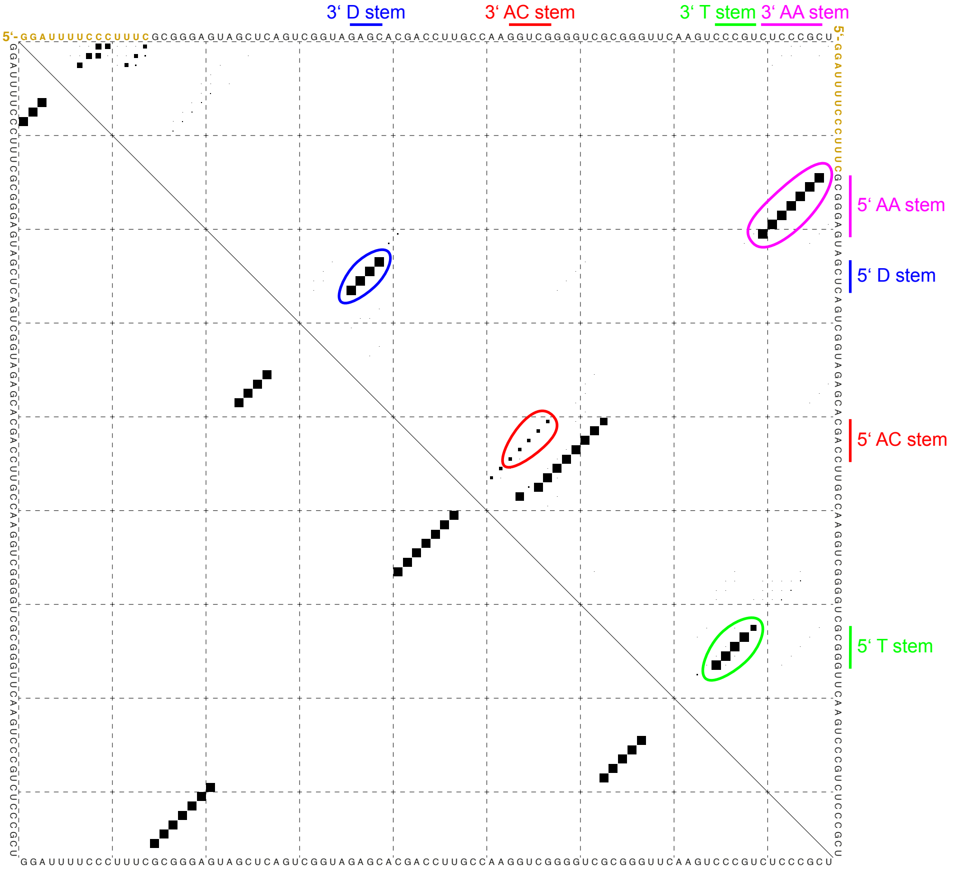

3‘ D stem
3‘ AC stem
3‘ T stem
3‘ AA stem
5‘ AA stem
5‘ D stem
5‘ AC stem
5‘ T stem

B

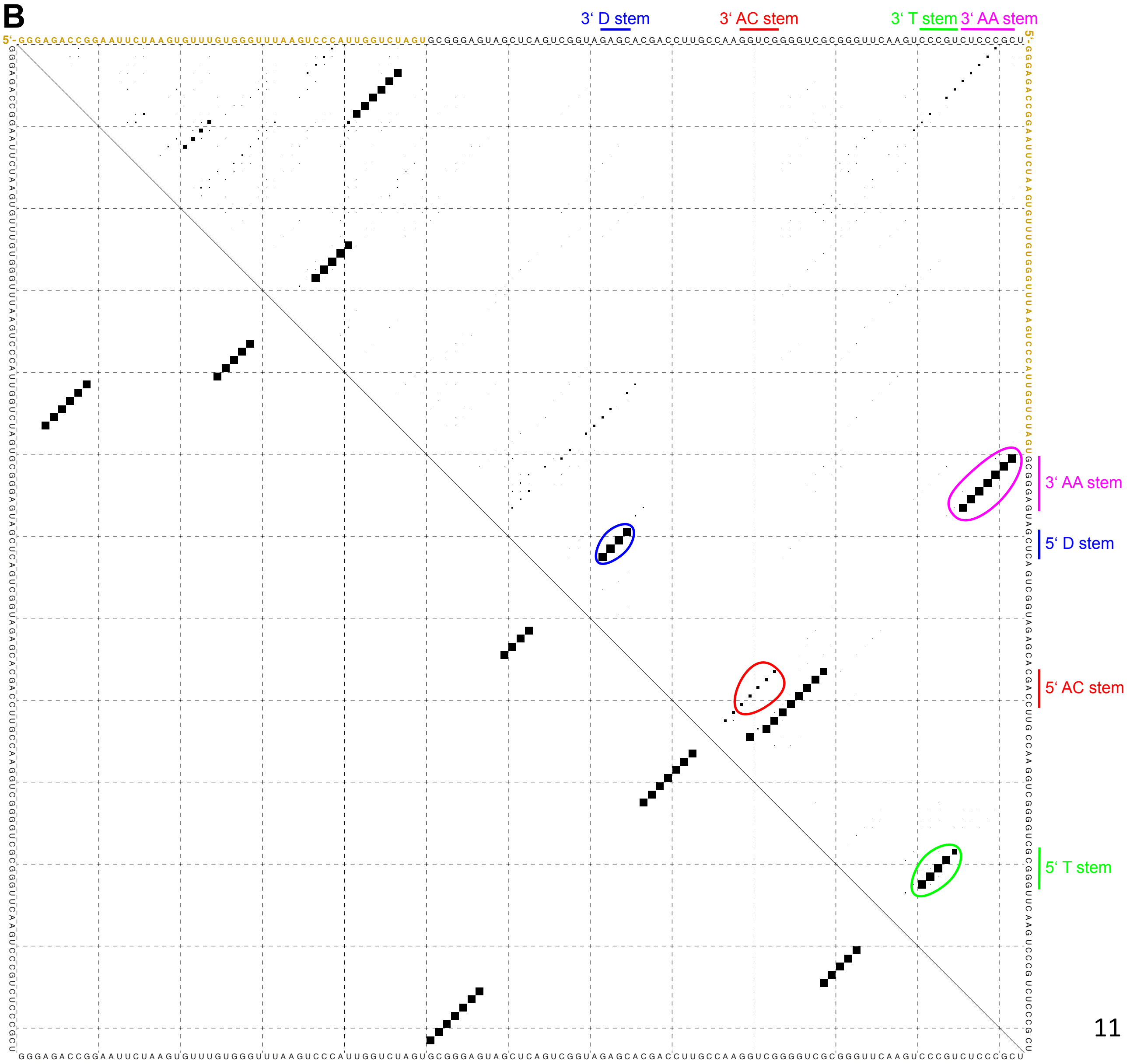

3‘ D stem
3‘ AC stem
3‘ T stem
3‘ AA stem
3‘ AA stem
5‘ D stem
5‘ AC stem
5‘ T stem

C

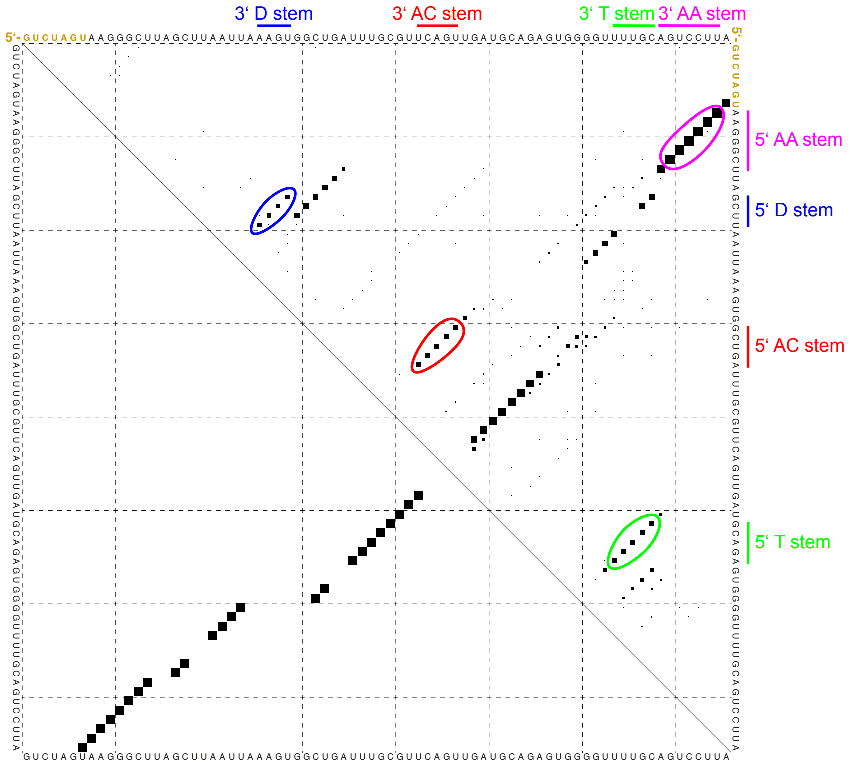

3‘ D stem
3‘ AC stem
3‘ T stem
3‘ AA stem
5‘ AA stem
5‘ D stem
5‘ AC stem
5‘ T stem

D

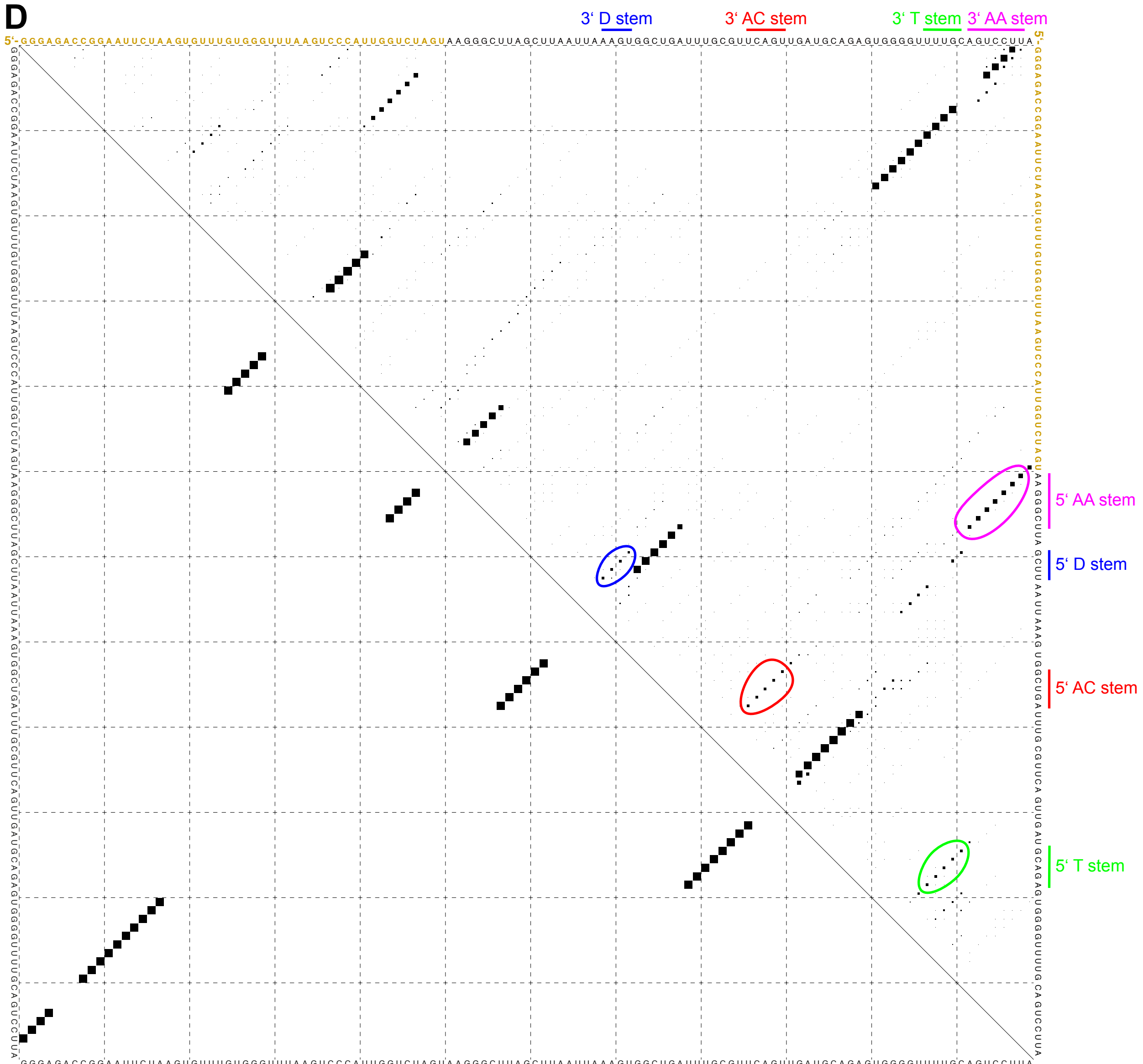

3‘ D stem
3‘ AC stem
3‘ T stem
3‘ AA stem
5‘ AA stem
5‘ D stem
5‘ AC stem
5‘ T stem

Supplementary Figure S7. Dot plots of RNA secondary structure predictions by *RNAfold* (2) for *Tt* pre-tRNA^{Gly} with (**A**) its 14-nt leader or with (**B**) the 50-nt leader of mitochondrial pre-tRNA^{Ala} (see also Figure 1 and Table 2), and mitochondrial pre-tRNA^{Ala} carrying (**C**) a 7-nt or (**D**) a 50-nt leader (see also Supplementary Figure S1 and Table S3); all sequences end with the discriminator position +73. The dots representing the base pairs of the four tRNA stems (AA, aminoacyl acceptor; AC, anticodon) are encircled and colored as in Figure 1 and Supplementary Figure S1; the 5'-leader nucleotides are colored brown. The dot pattern below the diagonal represents the minimum free energy (MFE) structure, whereas that above the diagonal illustrates the diversity of the ensemble of predicted near-isoenergetic structures.

The dot plot analysis indicates that the representation of the cloverleaf structure of the stable bacterial tRNA^{Gly} within the folding ensemble is largely independent of leader length (note that *RNAfold* favors, leader-independent, the formation of an alternative extended stem involving anticodon arm and variable loop nucleotides in the case of *Tt* tRNA^{Gly}). In comparison, it is evident for the structurally more labile human mitochondrial pre-tRNA^{Ala} with a 50-nt leader (compared with a 7-nt leader) that folding variants involving sequence elements of the leader and tRNA body come up while variants representing the tRNA cloverleaf are reduced. This suggests that, depending on the respective tRNA, long leaders may interfere with tRNA folding equilibria.

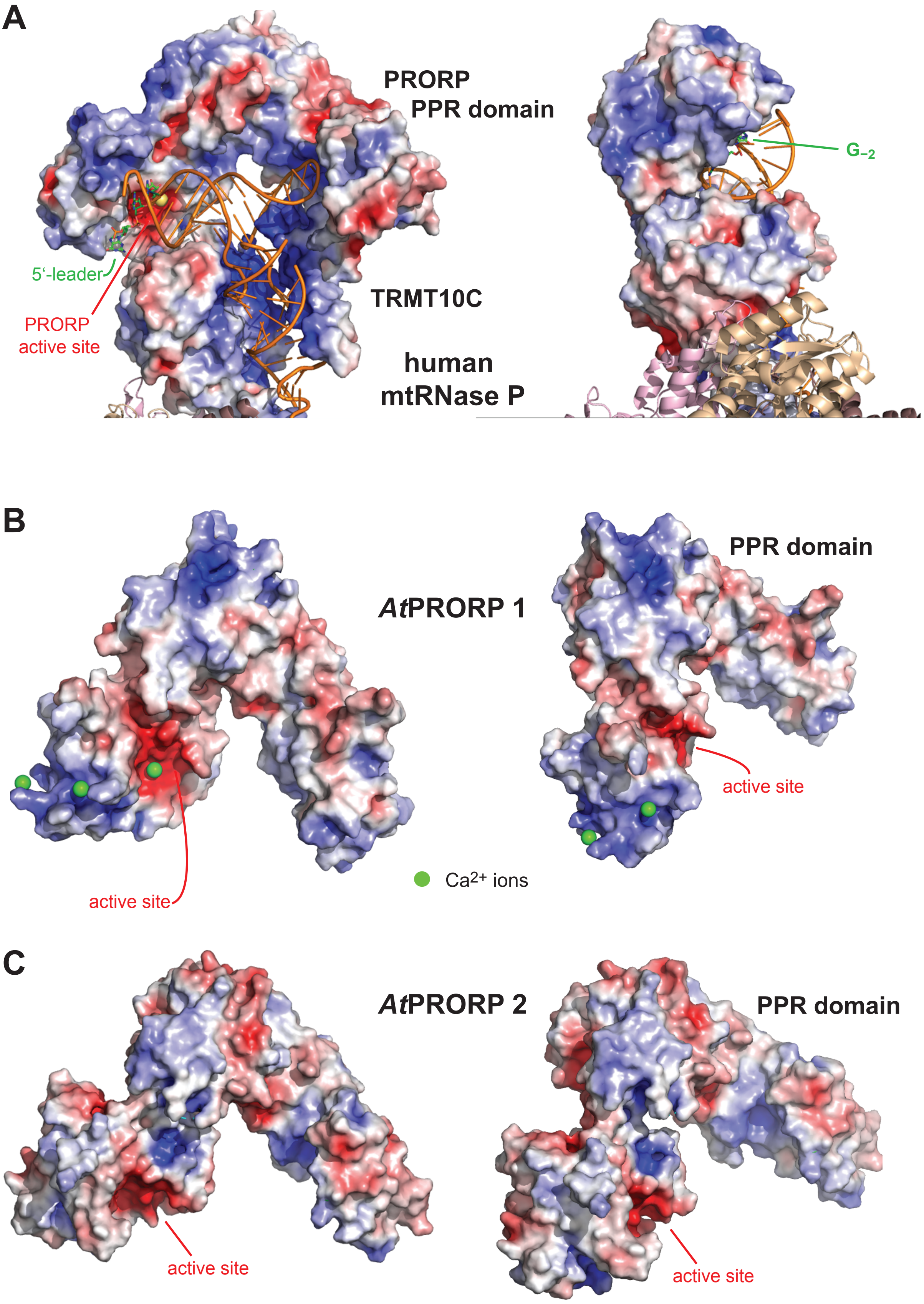
A
PRORP
PPR domain
G$_{-2}$
5'-leader
PRORP
active site
TRMT10C
human
mtRNase P
B
PPR domain
*At*PRORP 1
active site
active site
Ca^{2+} ions
C
*At*PRORP 2
PPR domain
active site
active site

Supplementary Figure S8. Electrostatic surface potential of (**A**) human PRORP (PDB 7ONU), (**B**) *At*PRORP1 (PDB 4G26) and (**C**) *At*PRORP2 (PDB 5FT9) generated with PyMOL (Schrödinger). (**A**) Surface potential of human PRORP and TRMT10C in two different orientations. SDR5C1 subunits and $tRNA^{Tyr}$ are shown as ribbon cartoons. The PRORP active site and pre-tRNA nt G_{-2}, A_{-1} und G_{+1} (sticks depiction) are indicated on the left, G_{-2} is marked in the orientation on the right; yellow sphere: Mg^{2+} in the active site. (**B**, **C**) Surface potential of (**B**) *At*PRORP1 and (**C**) *At*PRORP2, illustrated in two orientations for each protein. Color code of surface potentials: red, negatively charged amino acid side chains; white, uncharged side chains; blue, positively charged side chains.

Supplementary References

1. Sprinzl, M., Horn, C., Brown, M., Ioudovitch, A. and Steinberg, S. (1998) Compilation of tRNA sequences and sequences of tRNA genes. Nucleic Acids Res., 26, 148-153.
2. Lorenz, R., Bernhart, S.H., Höner Zu Siederdissen, C., Tafer, H., Flamm, C., Stadler, P.F. and Hofacker, I.L. (2011) ViennaRNA Package 2.0. Algorithms Mol Biol, 6, 26.
